# Integrated bioinformatics analysis of SEMA3C in tongue squamous cell carcinoma using machine-learning strategies

**DOI:** 10.1186/s12935-024-03247-y

**Published:** 2024-02-06

**Authors:** Huixin Dou, Can Song, Xiaoyan Wang, Zhien Feng, Yingying Su, Hao Wang

**Affiliations:** 1 Department of Stomatology, Beijing Tiantan Hospital, Capital Medical University, Beijing, 100070 China; 2 Research and Development Department, Allife Medicine Inc., Beijing, China; 3 Beijing Laboratory of Oral Health, Capital Medical University, Beijing, China; 4 Department of Biochemistry and Molecular Biology, School of Basic Medicine, Capital Medical University, Beijing, China; 5 Department of Oral and Maxillofacial & Head and Neck Oncology, Beijing Stomatological Hospital, Capital Medical University, Beijing, China

**Keywords:** Tongue squamous cell carcinoma, SEMA3C, Biomarkers, Integrated bioinformatics analysis, Prognosis

## Abstract

Tongue squamous cell carcinoma (TSCC) is an aggressive oral cancer with a high incidence of metastasis and poor prognosis. We aim to identify and verify potential biomarkers for TSCC using bioinformatics analysis. To begin with, we examined clinical and RNA expression information of individuals with TSCC from the Gene Expression Omnibus (GEO) database. Differential expression analysis and functional analysis were conducted. Multiple machine-learning strategies were next employed to screen and determine the hub gene, and receiver operating characteristic (ROC) analysis was used to assess diagnostic value. Semaphorin3C (SEMA3C) was identified as a critical biomarker, presenting high diagnostic accuracy for TSCC. In the validation cohorts, SEMA3C exhibited high expression levels in TSCC. The high expression of SEMA3C was a poor prognostic factor in TSCC by the Kaplan–Meier curve. Based on the Gene Ontology (GO) analysis, SEMA3C was mapped in terms related to cell adhesion, positive regulation of JAK-STAT, positive regulation of stem cell maintenance, and positive regulation of NF-κB activity. Single-cell RNA sequencing (ScRNA-seq) analysis showed cells expressing SEMA3C were predominantly tumor cells. Then, we further verified that SEMA3C had high expression in TSCC clinical samples. In addition, the knockdown of SEMA3C suppressed the proliferation, migration, and invasion of TSCC cells in vitro. This study is the first to report the involvement of SEMA3C in TSCC, suggesting that upregulated SEMA3C could be a novel and critical potential biomarker for future predictive diagnostics, prevention, prognostic assessment, and personalized medical services in TSCC.

**Supplementary Information:**

The online version contains supplementary material available at 10.1186/s12935-024-03247-y.

## Introduction

Tongue squamous cell carcinoma (TSCC) is a prevalent malignant tumor of the oral cavity, accounting for approximately 350,000 new cases globally each year [1]. TSCC is clinically aggressive, has a high recurrence rate, and has a lower survival rate than general head and neck cancer [2, 3], with a five-year survival rate of less than 60% [4].

Currently, surgical resection, chemotherapy, and radiation are the most commonly used strategy in TSCC therapy. However, these treatments are unsatisfactory. The lack of therapeutic efficacy is mainly because most of the patients seeking treatment are in intermediate to advanced stages with limited therapeutic options. This dysfunction has a substantial impact on life quality, as well as a poor prognosis [5, 6]. Secondly, current screening methods are not sufficiently precise to allow early intervention and prognostic assessment [7]. In recent years, bioinformatics combined with machine learning has been used in various healthcare fields for predicting, preventing, diagnosing, and assessing prognosis. Models using machine learning appear more accurate than traditional screening methods as they analyze massive data and, through algorithms, identify patterns for making predictions and guiding personalized management [8, 9].

Machine learning (ML) methods such as weighted gene co-expression network analysis (WGCNA), least absolute shrinkage and selection operator (LASSO) logistic regression, and random forests (RF) are used to identify new prognostic and predictive factors in a wide range of diseases such as head and neck squamous cancer [10], colon cancer [11], liver cancer [12], breast cancer [13], and Alzheimer’s disease [14]. This approach is also utilized to discover potential treatments for this highly complex disease.

Semaphorins (SEMAs) are secreted membrane signaling proteins that are indispensable in the development and maintenance of many organs and tissues. They have also been implicated in the regulation of various cancer processes, including angiogenesis, cancer cell invasion and metastasis, tumor growth, and cancer cell survival [15]. Previous studies have found that SEMA3C is associated with tumor progression in various types of cancers, including prostate [16], pancreatic [17], brain [18], breast [19], cervical [20], and gastric [21] cancers. Its expression in neoplastic and cancerous tissues is higher than that in normal epithelial tissues, and the high expression of SEMA3C correlates with poor prognosis. Moreover, inhibition or knockdown of SEMA3C suppresses tumor cell proliferation, and colony formation ability in vitro, and inhibits tumor growth in vivo. Chen [22] and Zhang et al.  [23] found that SEMA3C shows worse disease‐free survival with higher expression and is up‐regulated in clinical samples of head and neck squamous cell carcinoma (HNSCC). The discovery is of utmost significance and provides the basis for subsequent investigations into SEMA3C in HNSCC. However, no previous studies have reported an association between SEMA3C and TSCC. Therefore, our study provides supplementary evidence regarding the association between TSCC and SEMA3C.

In this study, we obtained a microarray dataset of TSCC from the GEO through an integrated bioinformatics approach and machine learning strategies for analysis to screen and identify key biomarker genes for TSCC, offering prospective goals for TSCC diagnosis, prevention, prognostic assessment, and customized treatment.

## Methods and materials

### Dataset collection and processing

The original datasets GSE31056 and GSE34105 were downloaded from the NCBI-GEO database (http://www.ncbi.nlm.nih.gov/geo/) [24], which is an international public repository. GSE31056 [25] contains 23 oral carcinoma samples, 49 histologically normal margin samples, and 24 adjacent normal tissue samples, established on the GPL10526 platform. GSE34105 [26] contains 62 TSCC samples and 16 non-malignant control samples, established on the GPL14951 platform. GSE13601 [27], GSE160042, and GSE138206 were used as validation sets to verify the expression of the hub gene. Furthermore, scRNA-seq data GSE172577 was used to profile the cell populations expressing key biomarkers in TSCC. The clinical information of these datasets is summarized in Additional file 1: Table S1. The datasets used in this study are all TSCC-related samples and the selected datasets have sufficient sample size. Multiple datasets were chosen mainly to allow for cross-validation and thus to draw more definitive conclusions.

### Identification of differentially expressed genes

The “limma” V3.46.0 package in the R software was used to screen for differentially expressed genes (DEGs) in TSCC and normal tissues. The criteria for screening DEGs were |Log2 fold-change (log2 FC)|≥ 1 and adjusted P-value < 0.05. The ggplot2 V3.3.5 package of the R software was used to generate the volcano map, the Pheatmap V1.0.12 package of the R software was used to plot the heat map of the first 30 DEGs in each dataset, and the Venny 2.1.0 online tool (https://bioinfogp.cnb.csic.es/tools/venny/index.html) was used to plot the VennDiagram.

### Functional enrichment analysis of DEGs

Gene function and metabolic pathway analyses were performed on the intersecting DEGs of the datasets using the clusterProfiler V3.18.1 package and Goplot V1.0.2 package in the R software (significant as a P < 0.05 and a q-value < 0.05). The functional enrichment analysis included GO analysis and Kyoto Encyclopedia of Genes and Genomes (KEGG) pathway analysis.

### Screening and validation of hub genes

WGCNA, LASSO, and RF were used to screen key markers for TSCC in this study. WGCNA is a powerful method for exploring the complex relationships between gene expression profiles and phenotypes. It can greatly narrow down the range of genes to be screened, thereby increasing the accuracy of pinpointing genes associated with key traits [28]. LASSO regression is based on the regression method, which reduces the complexity of the model, prevents overfitting, and utilizes a smaller sample size to effectively filter features, resulting in a more accurate prediction algorithm [29]. RF algorithms are more accurate and well-suited for analyzing complex data, such as omics data, which is often high-dimensional [30]. The R software packages used for implementing the different classification models were as follows: randomForest V4.7-1 package for the RF model, glmnet V4.1-4 package for LASSO logistic regression analysis, and WGCNA V1.71 package for conducting WGCNA. To conduct further analysis, the genes that overlapped among the three models were identified. The diagnostic worth of the TSCC hub gene was assessed by computing ROC curves and the area under the curve (AUC) using the pROC V1.18.0 package [31] of R software. Statistical significance was set at a two-sided P-value of less than 0.05. The expression of the hub gene in TSCC and control was evaluated by combining the validation datasets GSE13601, GSE160042, and GSE138206 with GSE31056.

### Survival and statistical analysis

For survival analysis, the data in the GSE31056 and GSE41613 datasets were collated, and gene expression values were grouped by median into low and high-expression groups. Survival curves were plotted according to the Kaplan–Meier estimation method, and Cox proportional hazards models were used to estimate hazard ratios (HR) and 95% confidence intervals (CI) using GraphPad Prism (Version 8.1.1). P < 0.05 was considered to indicate statistically significant differences.

### Functional enrichment analysis of the hub gene

Using the datasets GSE31056 and GSE41613, Pearson correlation analysis between the hub gene and all other genes was performed using R software. Common genes with a Pearson correlation analysis result of P < 0.05 were selected from both datasets. Subsequently, GO analysis was conducted using the DAVID online tool (DAVID Functional Annotation Bioinformatics Microarray Analysis (ncifcrf.gov)).

### Single‑cell RNA sequencing data analysis

The preprocessed data of GSE172577 were obtained from the GEO database. Cellranger (10X Genomics) was used for preprocessing. After downloading the data, they were imported into R and analyzed using the Seurat V4.1.0 package.

Initially, quality control was performed to filter out cells that did not meet specific criteria. These criteria included a gene count per cell greater than 200 and less than 7500, as well as a percentage of mitochondrial genes below 20%.

Next, the data were normalized using the NormalizeData function. To identify the most variably expressed genes for downstream analysis, the top-ranked 2000 genes were selected using the “vst” method in the FindVariableFeatures function. Before conducting principal components analysis (PCA), the data were scaled using the ScaleData function.

PCA, cluster analysis, and Uniform Manifold Approximation and Projection (UMAP) dimensional reduction were then applied using the RunPCA, FindClusters, and RunUMAP functions, respectively. The resulting cell clusters were visualized using UMAP plots generated by the DimPlot function. Bubble plots were generated by the ggplot2 V3.3.5 package of the R software, and the line graph was drawn in Excel.

### Human TSCC samples

To validate the expression of key biomarkers in human TSCC specimens, TSCC tissues and their paired adjacent normal tissues (> 2 cm from the tumor edge) were collected from 9 patients undergoing maxillofacial surgery at Beijing Stomatology Hospital. None of the patients had received chemotherapy or radiation therapy before surgery. Six of the nine pairs of tissue specimens were randomly selected for immunohistochemical staining. The mRNA expression of the remaining 3 pairs was verified by quantitative real-time polymerase chain reaction (qRT-PCR). Written informed consent was obtained from all participants, and the collection protocol for human tissue specimens was approved by the Research Ethics Committee of Capital Medical University, conducted in compliance with the Declaration of Helsinki, and approved by the Prospective, Observational, Real-world Oral Malignant Tumors Study (approval number: NCT02395367).

### Immunohistochemistry (IHC)

The deparaffinization of TSCC and paracancerous specimens was conducted by immersing them in xylene, followed by rehydration in a series of graded alcohol. To block endogenous peroxidase activity, a 3% H_2_O_2_ solution was applied. Antigen retrieval was achieved through heating in citrate buffer. The sections were then subjected to overnight incubation at 4 °C with a primary antibody against SEMA3C (1:100 dilution, PA5-103168, Thermo Fisher Scientific, USA). A horseradish peroxidase-conjugated secondary antibody was subsequently added and incubated at room temperature for 30 min. The color reaction was developed using 3,3′-diaminobenzidine (DAB) solution (Dako, Denmark).

### Cell culture and transfection of SCC25 and SCC9 with shSEMA3C

Human tongue squamous cell carcinoma SCC25 and SCC9 cells were purchased from Sunncell (China) and BeNa Culture Collection (BNCC, China), respectively. Cultures were carried out at 37 °C and 5% CO_2_ in Dulbecco’s Modified Eagle Medium/Ham’s F 12 nutrient (DMEM/F12, Gibco, USA) supplemented with 10% heat-inactivated fetal bovine serum (FBS, Gibco, USA). The shRNA used was SEMA3C shRNA (5′-GCCAAGATCAACTTCAAAGTT-3′) and the control shRNA was purchased from Xitubio Biotechnology Co., Ltd (China). Cells were infected with shSEMA3C lentivirus with a final concentration of 10 µg/ml according to the polybrene instruction (Sigma-Aldrich, USA). Then, these cells were utilized for further experiments for 48 h. The stable transfectants were selected with 1 µg/ml puromycin. Seven days after the addition of puromycin, completion of selection was confirmed by the absence of live cells in the control nontransfected culture with puromycin.

### RNA extraction and qRT-PCR

Total RNA was extracted from tissues or cells with TRIzol (Invitrogen, USA), according to the manufacturer’s instructions. Total RNA was reverse transcribed using a reverse transcription kit (TransGen Biotech, China), according to the manufacturer’s protocol. The TransStart® Top Green qPCR SuperMix Kit (TransGen Biotech, China) was used for qRT-PCR. GAPDH was used to normalize the RNA levels using the 2^−ΔΔCt^ method. The sequences of the primers for qRT-PCR were: SEMA3C-F: 5′-TAA CCA AGA GGA ATG CGG TCA-3′; SEMA3C-R: 5′-TGC TCC TGT TAT TGT CAG TCA GT-3′; GAPDH-F: 5′- GGA GCG AGA TCC CTC CAA AAT-3′; and GAPDH-R: 5′- GGC TGT TGT CAT ACT TCT CAT GG-3′.

### CCK-8 assay

Cell growth was measured using a CCK-8 kit (Sigma-Aldrich, USA) according to the instructions. Briefly, approximately 5 × 10^3^ cells per well were plated into 96-well plates and cultured in the indicated medium. Cell proliferation, measured at 450 nm, was examined every day for three days according to the manufacturer’s protocol. All experiments were performed three times.

### Colony formation assay

2 × 10^3^ cells per well were seeded in 6-well plates, and the culture was terminated when the cells formed obvious clones under the microscope. The colonies were fixed with 4% paraformaldehyde and stained with 0.1% crystal violet (Sigma-Aldrich, USA) for 15 min at room temperature. Finally, the colony number was quantified.

### Wound healing assay

SCC9 and SCC25 were plated into 6-well plates (about 4 × 10^5^ cells/well). When the confluence reached 100%, a scratch was made using a pipette tip of 200 µl. The detached cells were removed with serum-free medium. At 0 h, 24 h, and 48 h, the wounded area was photographed. Migration rate was measured as follows: % migration rate = ((0 h wound area − 24 h/48 h wound area) × 100)/0 h wound area.

### Transwell experiment

SCC9 and SCC25 cells were loaded in a prepared Transwell chamber (8 um pore size; Corning Inc., USA) with Matrigel (BD Biosciences, USA), and the cell density of each group was adjusted with serum-free DMEM/F12 culture medium. Briefly, 1 × 10^4^ cells were added to the upper chamber, and DMEM/F12 culture medium containing 10% FBS was added to the lower chamber. They were incubated for 48 h in 5% CO_2_ at 37 ℃. Non-invading cells and the matrigel layer were mechanically removed using cotton swabs, and the microporous membrane was fixed with 4% paraformaldehyde and stained with 0.1% crystal violet (Sigma-Aldrich, USA) for 15 min at room temperature. The number of transmembrane cells from 5 randomly different fields was selected and photographed from every group.

### Statistical analysis

Unpaired t-tests (also known as independent t-tests) are used to compare the means/averages of two independent or unrelated groups to determine if there is a significant difference between them. In this study, comparisons were conducted using the unpaired t-test. Statistical analysis and the graphs were performed using GraphPad Prism 8 software. The Kaplan–Meier and log-rank tests are extensively employed in survival analysis. The Kaplan–Meier curve is employed to illustrate the association between survival time and survival probability, while the log-rank test is a non-parametric statistical test used to compare the survival rates among two or more subgroups and determine if there exists a substantial difference in survival across these subgroups. Therefore, in this study, survival data were used to establish the Kaplan–Meier curves, and the differences among the groups were analyzed by the log-rank test. P-value < 0.05 was judged statistically significant.

## Results

### Identification of DEGs in TSCC and normal tissues

**Fig. 1:**
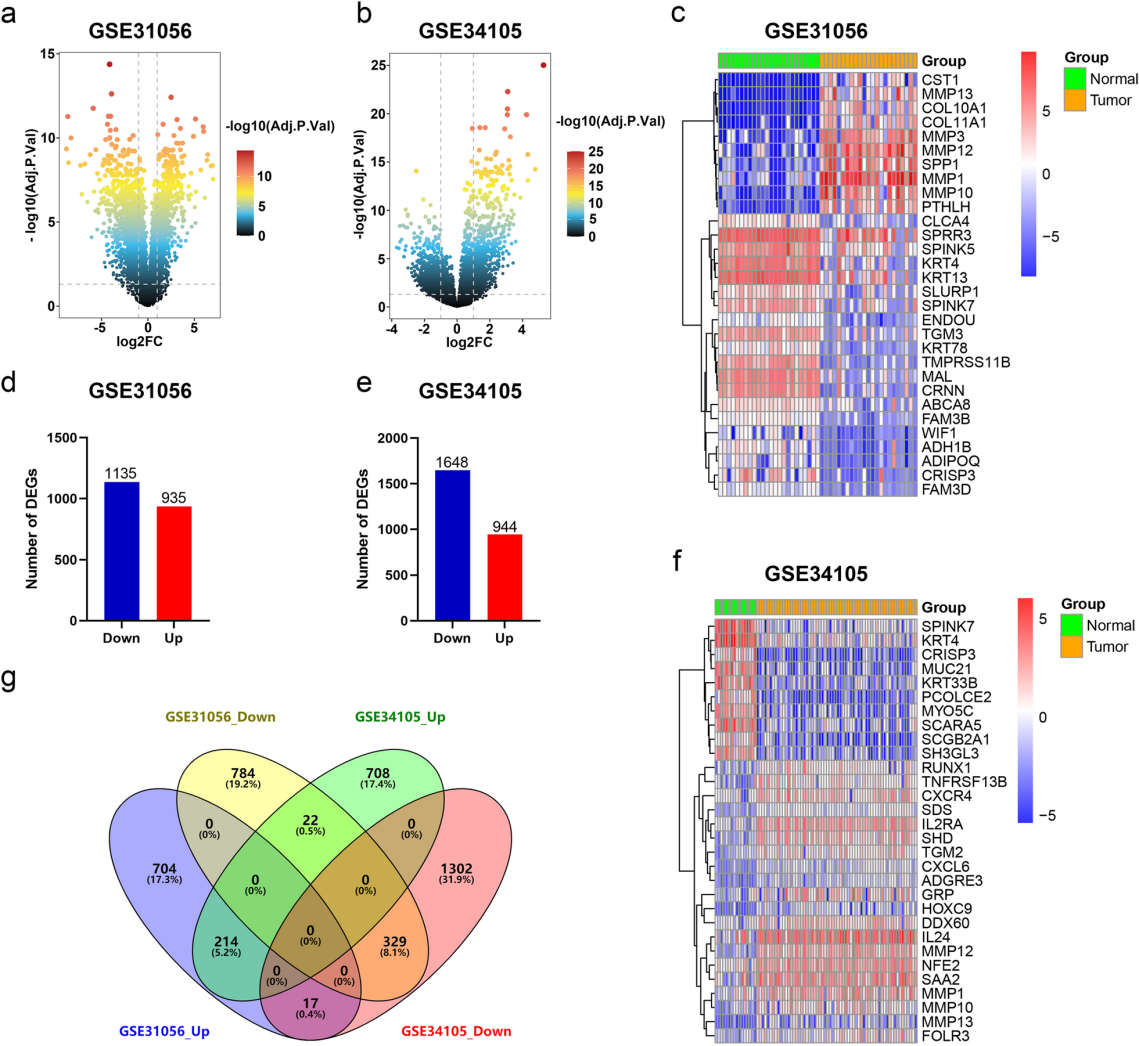
Identification of DEGs. **a**, **b** Volcano plots of DEGs distribution in GSE31056 (**a**) and GSE34105 (**b**). **c**, **f** Heatmaps of DEGs in GSE31056 (**c**) and GSE34105 (**f**). **d**, **e** Number of DEGs in GSE31056 (**d**) and GSE34105 (**e**). **g** Venn diagram of DEGs from the two datasets

In search of DEGs for TSCC and normal, the differential expression analysis was performed, and a total of 2070 genes were found to be differentially expressed in GSE31056, consisting of 935 upregulated genes and 1135 downregulated genes (Fig. 1a and d). In GSE34105, a total of 2592 genes were identified, including 944 upregulated genes and 1648 downregulated genes (Fig. 1b and e). Volcano plots were used to illustrate the distribution of these DEGs (Fig. 1a and b), and the top 30 DEGs, consisting of 10 upregulated genes and 20 downregulated genes in GSE31056, and 20 upregulated genes and 10 downregulated genes in GSE34105, were depicted in heatmaps (Fig. 1c and f). To determine the overlapping DEGs between the two datasets, a Venn analysis was performed, revealing 329 overlapping downregulated genes and 214 overlapping upregulated genes associated with TSCC (Fig. 1g, Additional file 1: Table S2).

### GO and KEGG pathway analyses of DEGs

**Fig. 2:**
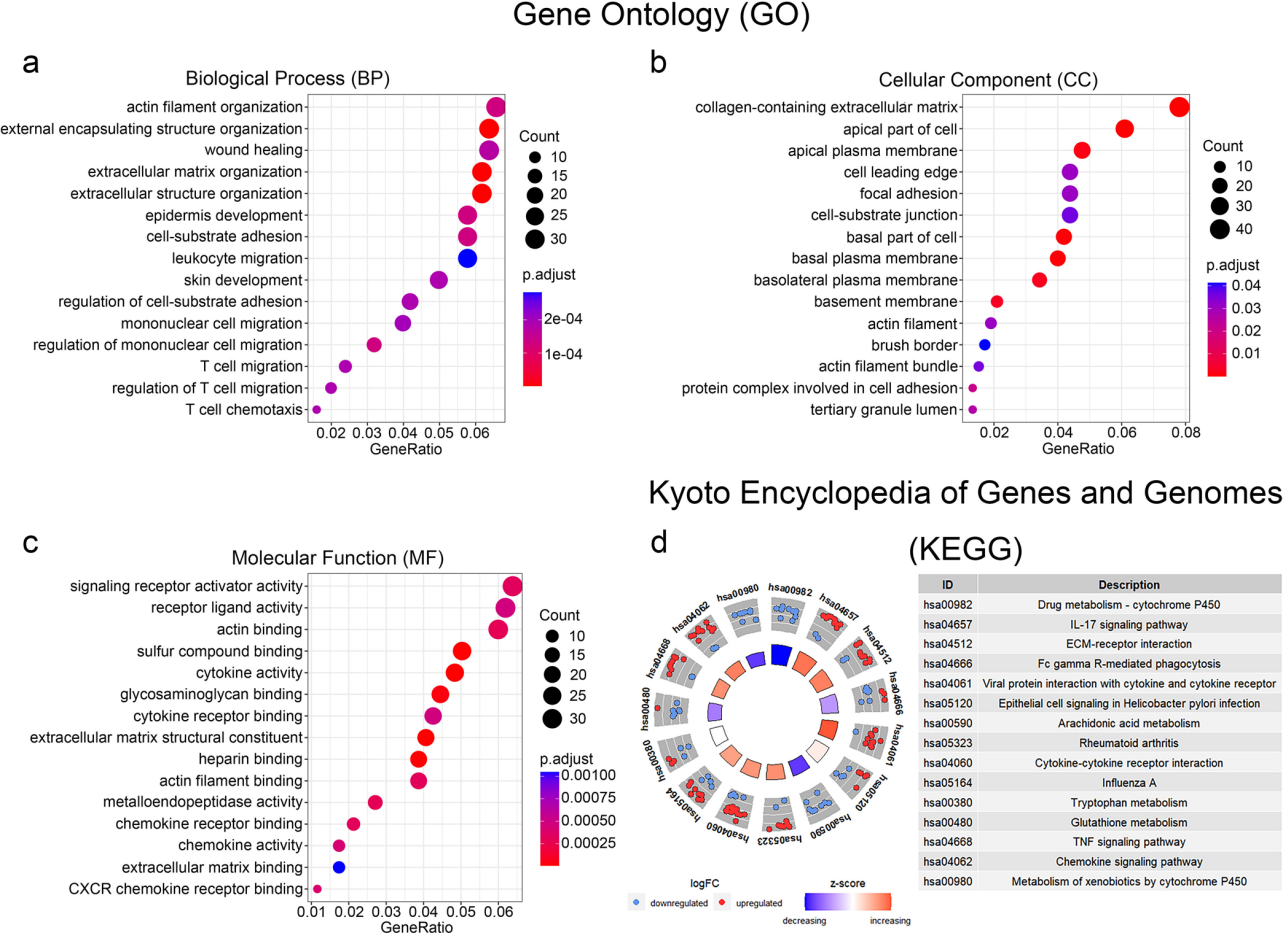
GO and KEGG pathway enrichment analyses of DEGs. **a**–**c** Bubble charts show GO-enriched items of DEGs in three functional groups: BP (**a**), CC (**b**), and MF (**c**). **d** Circle plot shows KEGG-enriched items of DEGs

In order to identify the biological functions of the above DEGs, GO and KEGG functional enrichment analyses were performed. GO analysis results showed that in terms of BP, actin filament organization, external encapsulating structure organization, wound healing, extracellular matrix organization, and extracellular structure organization were notably enriched (Fig. 2a). In terms of CC, collagen-containing extracellular matrix, apical part of cell, apical plasma membrane, cell leading edge, and focal adhesion were significantly enriched (Fig. 2b). In terms of MF, signaling receptor activator activity, receptor ligand activity, actin binding, sulfur compound binding, and cytokine activity were greatly enriched (Fig. 2c). Furthermore, KEGG pathway analysis was performed on the DEGs. The results revealed Drug metabolism-cytochrome P450, IL-17 signaling pathway, ECM-receptor interaction, cytokine–cytokine receptor interaction, and chemokine signaling pathway as the most highly enriched pathways (Fig. 2d). In conclusion, the DEGs were functionally related to the malignant progression of tumors, including proliferation, migration, and invasion.

### The dimensionality reduction analysis of the screened genes based on machine learning algorithms

**Fig. 3:**
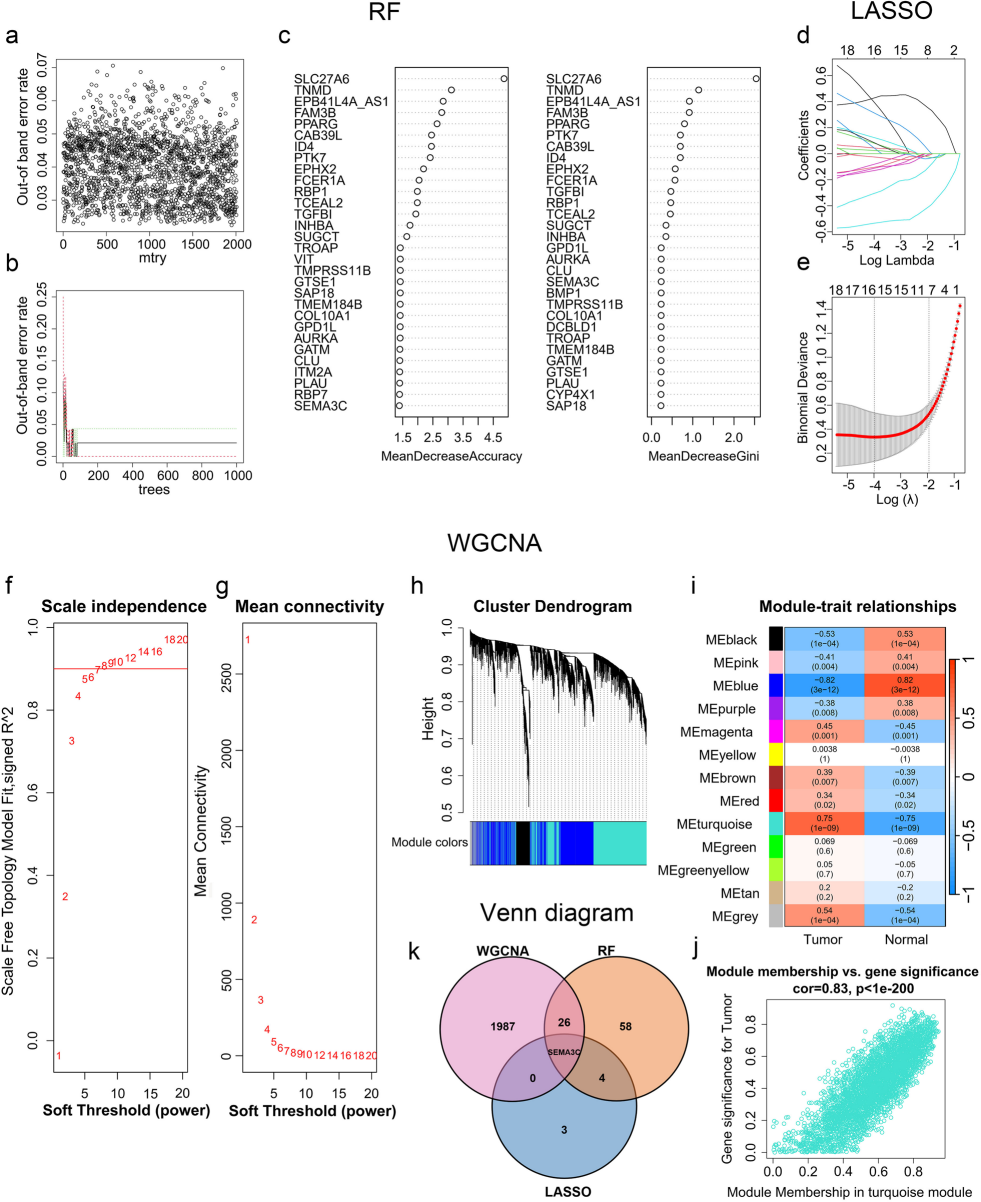
Screening of hub gene via multiple machine-learning algorithms. **a**–**c** Identification of hub gene by RF. Distribution of out-of-band (OOB) error rate at various values of mtry (**a**) and trees (**b**). Variable importance, as measured by the mean decrease in accuracy (left panel) or the Gini coefficient (right panel), is computed using the OOB error (**c**). Genes are shown in descending order of importance. **d**, **e** Establishment of hub gene by LASSO logistic regression analysis. LASSO coefficient profile of the 8 genes (**d**). Selection of the optimal parameter (lambda) in the LASSO model, and generation of a coefficient profile plot (**e**). **f**–**j** Process of WGCNA. Analysis of network topology for various soft-thresholding powers (**f**, **g**). The x-axis reflects the soft-thresholding power. The y-axis reflects the scale-free topology model fit index (**f**) and the mean connectivity (**g**). Clustering dendrogram of differentially expressed genes related to TSCC, with dissimilarity based on topological overlap, together with assigned module colors (**h**). Module-trait associations (**i**). The gene significance for TSCC in the turquoise module (**j**). **k** Venn diagram shows the intersection of the hub gene obtained by the three strategies

In this study, we applied three different algorithms, namely RF, LASSO logistic regression, and WGCNA, to identify critical marker genes. The results revealed that 89 genes were identified using the RF algorithm (Fig. 3a–c and Additional file 1: Table S3), while the LASSO logistic regression algorithm identified 8 genes (Fig. 3d and e and Additional file 1: Table S3). Furthermore, we employed the WGCNA analysis, using default-recommended parameters (Fig. 3f and g), to identify 13 notable co-expression modules (Fig. 3h). Our investigation of module-trait correlations showed that multiple modules were associated with TSCC (Fig. 3i). Among these modules, the turquoise module exhibited the most positive correlation with TSCC. Therefore, we focused on screening genes in the turquoise module and successfully identified 2014 genes (Fig. 3j and Additional file 1: Table S3). Our subsequent analysis using Venn diagrams revealed that SEMA3C was the overlapping gene identified by all three algorithms (Fig. 3k). Therefore, SEMA3C was defined as the best candidate gene for the TSCC marker and target.

### SEMA3C was a robust diagnostic marker for TSCC

**Fig. 4:**
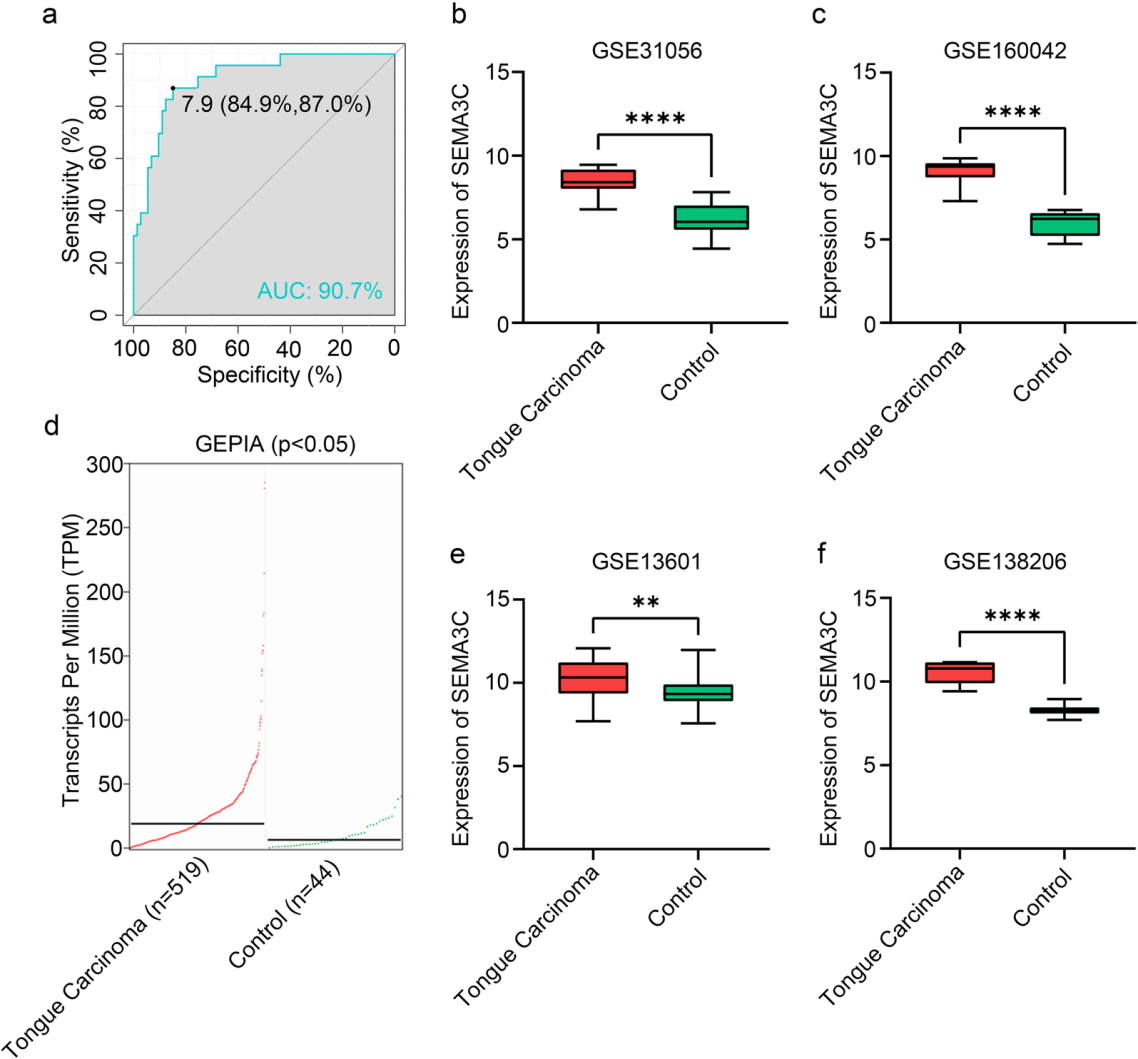
SEMA3C was highly enriched in TSCC. **a** The diagnostic power of SEMA3C in TSCC by ROC curve. **b**–**f** The expressions of SEMA3C in GSE31056 (**b**), GSE160042 (**c**), GSE13601 (**e**), GSE138206 (**f**), and GEPIA (**d**). (*P < 0.05, **P < 0.01, ***P < 0.001, ****P < 0.0001)

To evaluate the potential predictive value of the key gene marker in TSCC, ROC curves were generated. The results revealed that SEMA3C was a crucial gene with high accuracy in predicting the TSCC (AUC = 90.7%, Fig. 4a). We also examined the expression of SEMA3C in TSCC tissues and found that it was remarkably upregulated compared to controls in four different datasets, including GSE31056, GSE160042, GSE13601, and GSE138206 (all P < 0.01, Fig. 4b, c, e, f). Furthermore, the online tool GEPIA was used to validate that SEMA3C expression was significantly higher in TSCC than in the control group. (P < 0.05, Fig. 4d).

### Overexpression of SEMA3C was a prognostic risk factor in TSCC patients

**Fig. 5:**
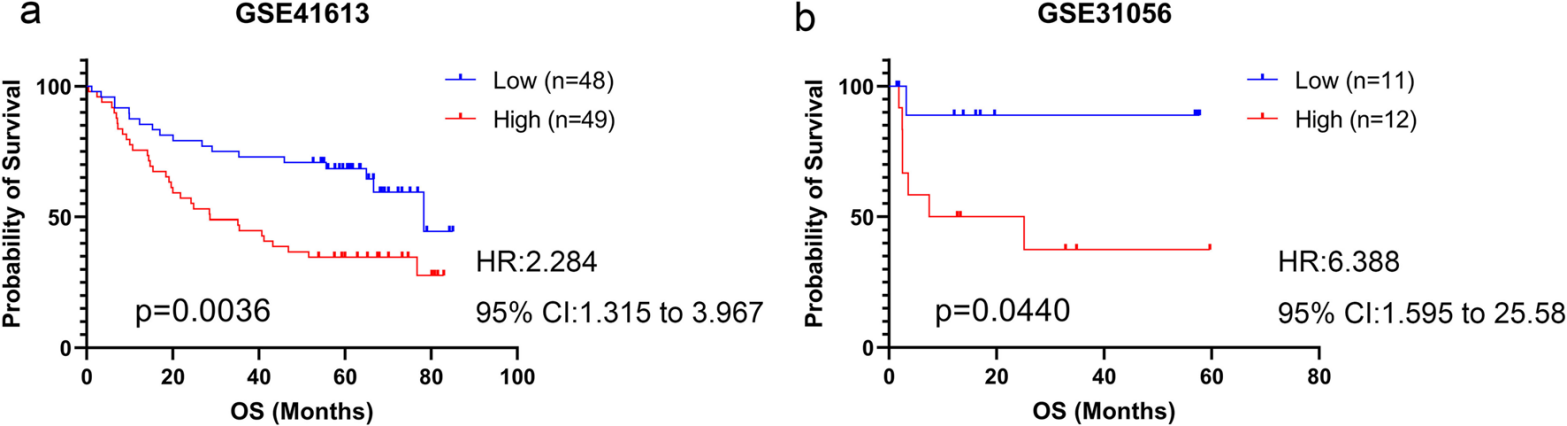
High SEMA3C expression as a poor prognostic factor in patients with TSCC. **a** Overall survival (OS) in the high and low SEMA3C groups in the GSE41613 cohort. **b** OS in the high and low SEMA3C groups in the GSE31056 cohort

Kaplan–Meier survival analysis was used to analyze the prognostic value of SEMA3C expression, and the results showed that high expression of SEMA3C was a poor prognostic factor in TSCC (P < 0.05) (Fig. 5a, b).

### Function enrichment analysis of the SEMA3C in TSCC

**Fig. 6:**
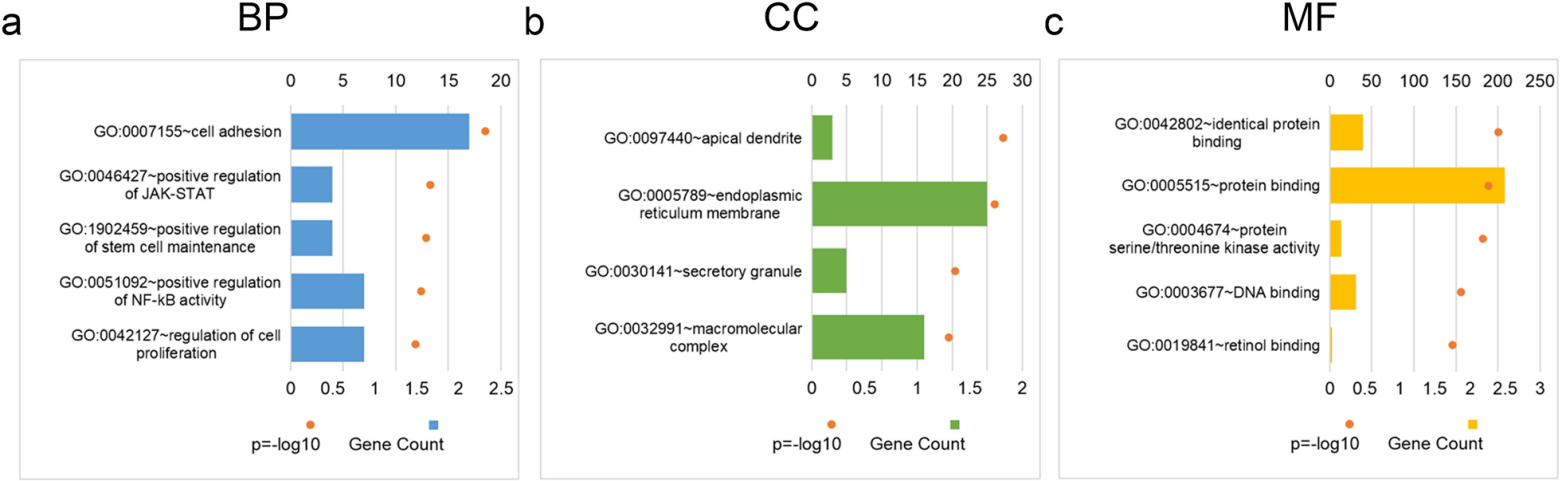
High expression SEMA3C promoted tumor process in TSCC. **a**–**c** GO-enriched items of SEMA3C in TSCC: BP (**a**), CC (**b**), and MF (**c**)

GO analysis was used to explore the oncogenic functions of SEMA3C. Firstly, we screened 309 genes correlated with SEMA3C from GSE31056 and GSE34105 datasets on NCBI (Additional file 1: Table S4). Then, the GO analysis suggested that genes correlated with SEMA3C were enriched in numerous cancer-related biofunctions such as cell adhesion, positive regulation of JAK-STAT, positive regulation of stem cell maintenance, and positive regulation of NF-κB activity, indicating that SEMA3C played a role in oncogenesis through these pathways (Fig. 6a–c).

### SEMA3C was specifically expressed in TSCC cells by scRNA‑seq analysis

**Fig. 7:**
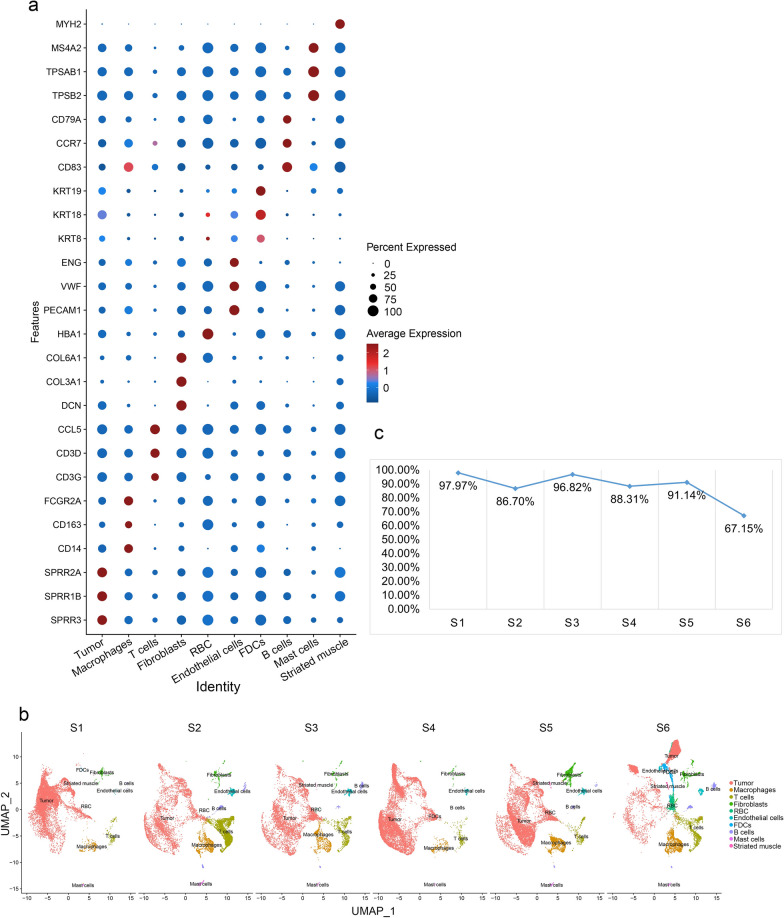
Single-cell RNA sequencing data analysis in TSCC tissues. **a** Expression of each group of cellular markers in different cells, demonstrating reliable cell definition. **b** Single cell cluster of 6 samples. **c** Percentage of tumor cells among cells expressing SEMA3C in 6 samples. SEMA3C was almost exclusively expressed in tumor cells, with low expression in other cells

To more accurately characterize the specific expression of SEMA3C in TSCC cells, we queried the scRNA-seq database to identify cell populations that express SEMA3C mRNA in TSCC. After implementing quality control and screening the cells, we performed unbiased clustering of the cells based on their gene expression profiles, with cell markers expressed in the corresponding cells and their expression relatively high (Fig. 7a), demonstrating that the cell definitions were reliable. The clustering identified 10 subpopulations, as shown in the UMAP plot (Fig. 7b). In TSCC cells, more than 90% of the SEMA3C-expressing cells were tumor cells (Fig. 7c), indicating that the treatment targeting SEMA3C may have little side effect.

### SEMA3C was specifically expressed in TSCC clinical samples

**Fig. 8:**
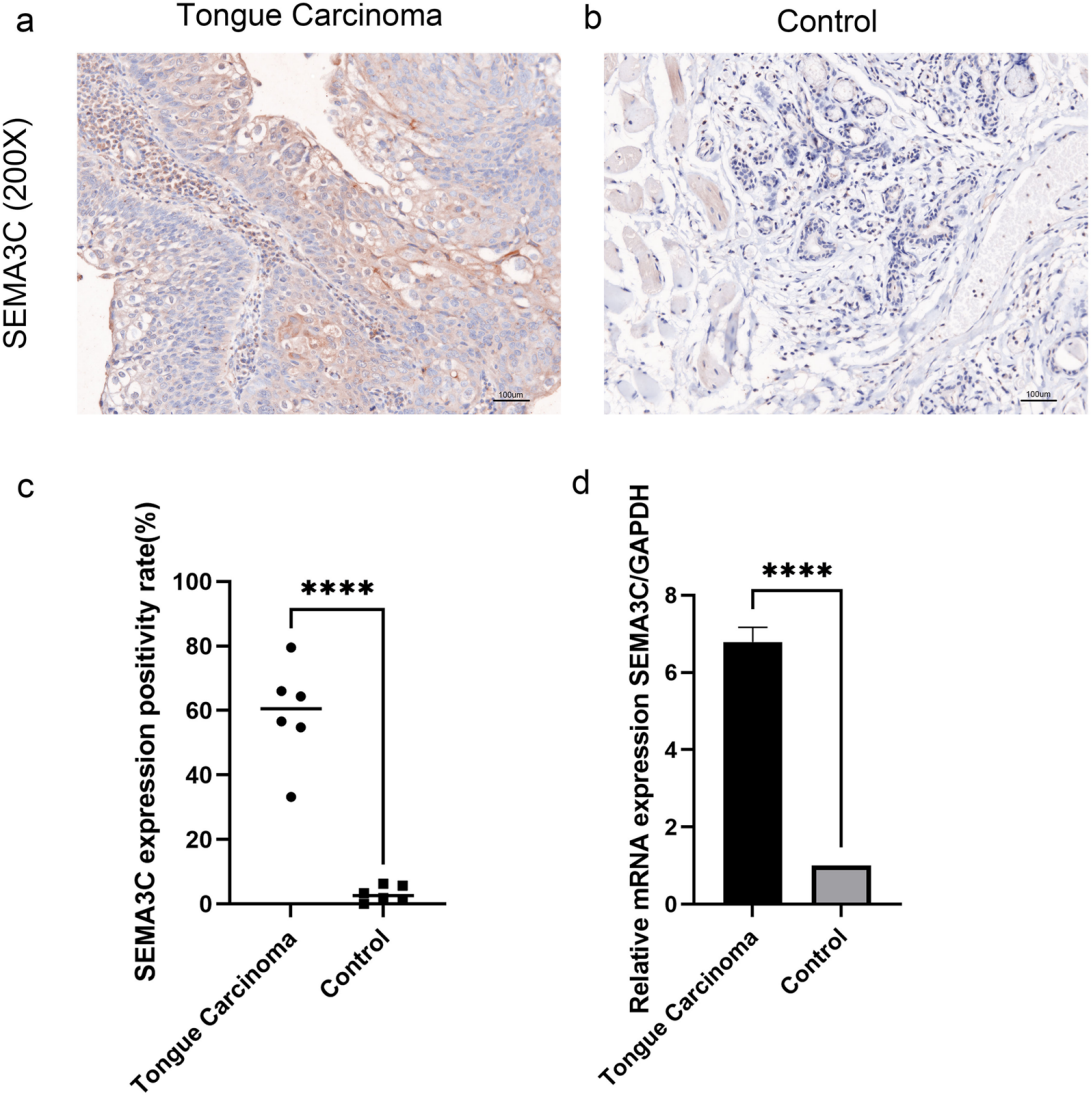
Expression of SEMA3C in human TSCC and normal tissues. **a**, **b** Immunohistochemistry for SEMA3C in TSCC (**a**) and normal (**b**) tissues from human. **c** Quantitative analysis of SEMA3C (n = 6, ****P < 0.0001). **d** Gene expression of SEMA3C was detected by qRT-PCR. Gene expression for SEMA3C was significantly higher in TSCC compared with normal tissues (****P < 0.0001)

As shown in Fig. 8a, b, c, IHC staining of SEMA3C in human specimens indicated significant positive staining in TSCC, while negative or low staining in normal tongue tissues. Moreover, consistent with IHC findings, qRT-PCR results revealed that the mRNA levels of SEMA3C in TSCC samples were highly upregulated compared with normal tongue samples (Fig. 8d). Above all, our findings demonstrated that SEMA3C might be a presumed oncogene driving the occurrence and development of TSCC.

### SEMA3C depletion inhibited cell proliferation and migration/invasion in TSCC cells

**Fig. 9:**
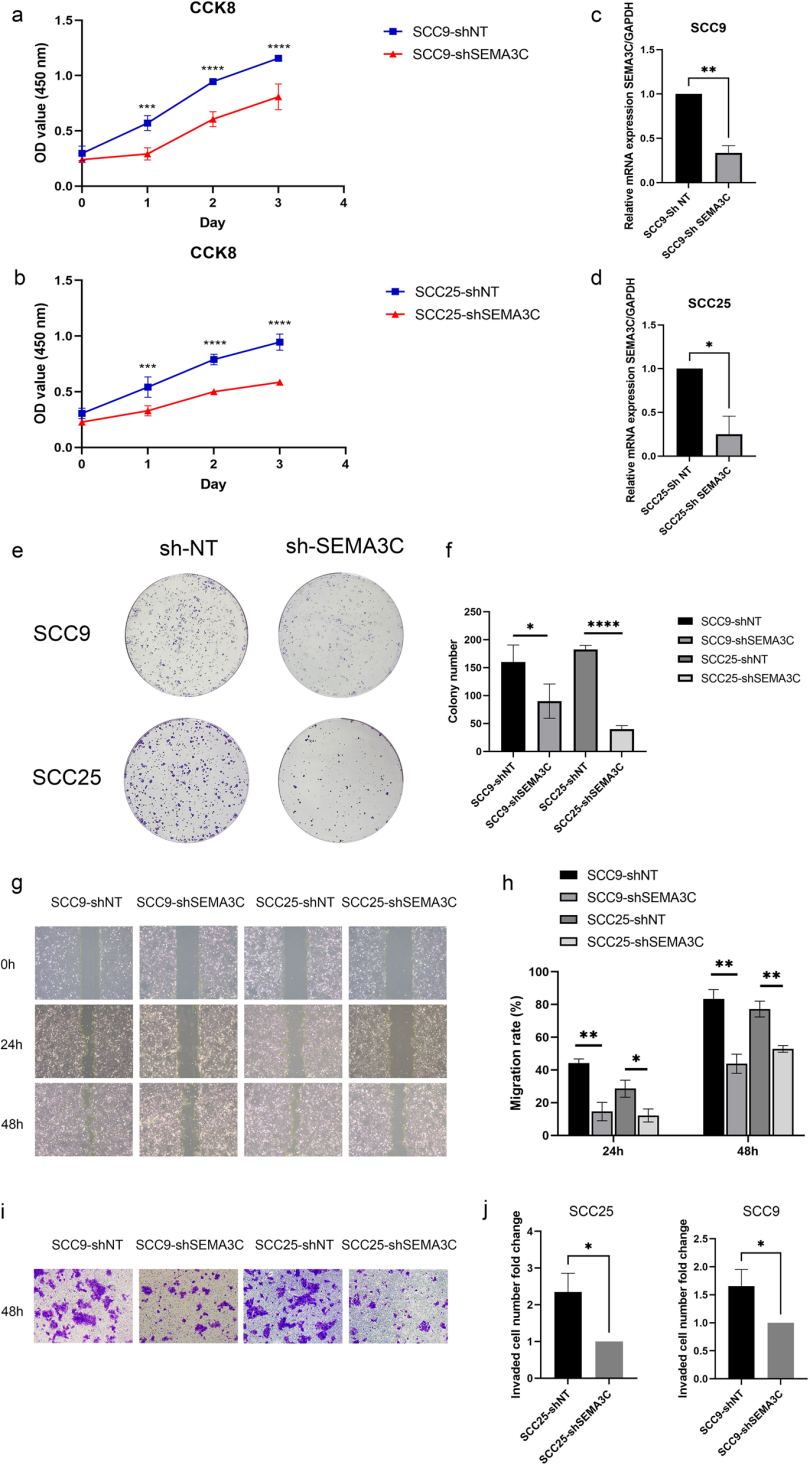
SEMA3C knockdown inhibited cell proliferation, migration, and invasion in TSCC cells. **c**, **d** qRT-PCR for the mRNA expressions of SEMA3C in SCC9 (**c**) and SCC25 (**d**) transfected with shRNA of SEMA3C. **a**, **b** Cell proliferation was remarkably suppressed when endogenous SEMA3C was silenced as measured by CCK-8 viability assay. **e**, **f** The potentials of colony formation were significantly inhibited in SEMA3C-depleted cells as compared to control (shNT). **g**–**j** The migration (**g**, **h**) and invasion (**i**, **j**) abilities were significantly reduced in shSEMA3C-transfected cells in wound healing and transwell assays, respectively. Data shown here were mean ± SD from three independent experiments, *P < 0.05, **P < 0.01, ***P < 0.001, ****P < 0.0001

We used shSEMA3C lentivirus and controlling lentivirus to stably transfect SCC9 and SCC25 cells. qRT-PCR demonstrated that SEMA3C mRNA expression was distinctly reduced following shRNA-SEMA3C transfection (Fig. 9c, d). CCK-8 viability assay and colony formation assay indicated a significantly lower proliferation rate in SCC9 and SCC25 cells following SEMA3C knockdown, compared to control cells (Fig. 9a, b, e, f). We then conducted a wound healing assay and transwell assay. Both wound healing and transwell invasion assays demonstrated the migratory and invasive abilities of cells following SEMA3C knockdown were significantly reduced (Fig. 9g–j). In vitro results indicated an important role of SEMA3C in promoting tumor progression by enhancing the capacities of proliferation, migration, and invasion.

## Discussion

The early detection, prevention, prognostic assessment, and intervention of TSCC are crucial in reducing the incidence and mortality rates and alleviating the substantial socioeconomic burden [32]. Screening for potential TSCC susceptibility gene markers and uncovering their underlying mechanisms are considered effective strategies. Our study showed that SEMA3C promoted TSCC cells growth, migration, and invasion. These results indicated that SEMA3C, as a hub gene for TSCC, might be involved in regulating the progression of TSCC and could be a potential molecular biomarker for accurate diagnosis in TSCC.

In this study, we conducted integrative bioinformatics analysis to identify 543 DEGs between TSCC and controls, and the functional enrichment analysis revealed the enrichment of terms related to growth, proliferation, migration, and invasion. Three machine-learning strategies, specifically WGCNA, and RF combined with LASSO, were employed to identify and determine the hub gene of TSCC. Bioinformatics analysis and machine learning strategies have been applied in oral squamous cell carcinoma, of which TSCC is one type, playing an important role in the diagnosis, prevention, and treatment of the disease. Aspirin has been demonstrated to inhibit the proliferation of oral squamous carcinoma using bioinformatics and in vitro studies, and it may be developed as an effective chemopreventive agent for the treatment of oral squamous carcinoma [33]. Differentially expressed genes and pathways with aberrant methylation are identified in oral squamous cell carcinoma by integrated bioinformatics analysis, that can help to reveal the underlying molecular mechanisms of oral squamous cell carcinoma [34]. Zhang et al. identified key modules and hub genes involved in the pathogenesis of OSCC by applying WGCNA to construct a gene co-expression network for the first time, and their study is of great significance for exploring the mechanism of OSCC tumourigenesis and searching for new prognostic biomarkers and therapeutic targets [35]. Compared with previous studies, the integrated procedure used in this study has the distinct advantage of selecting common markers shared by the combination of the three algorithms [28, 29], thus reducing the number of markers while significantly improving the specificity and sensitivity of the identification indicators. Notably, SEMA3C emerged as the most significant hub gene through this comprehensive approach, and the diagnostic efficacy of elevated SEMA3C in distinguishing individuals with TSCC from those without was evaluated using ROC analysis, which yielded high-accuracy results. It is important to highlight that the stable performance of SEMA3C expression was maintained across various validation datasets. These findings strongly support the notion that SEMA3C may potentially act as a causative factor in the onset of TSCC, thereby emphasizing its potential clinical utility in applications such as disease prediction, prevention, and personalized therapeutics.

Peacock et al. found that SEMA3C is a secreted soluble autocrine growth factor that promotes the growth of prostate cancer via Plexin B1- and NRP1‐mediated transactivation of EGFR, HER2/ErbB2, and MET signaling with activation of downstream signaling pathways such as SRC, MAPK, and PI3K/AKT pathways [16]. SEMA3C expression is higher in pancreatic cancer than in normal tissue, positively correlated with tumor stage, and inversely correlated with patient survival. In vitro experiments showed that SEMA3C promotes proliferation, invasion, and EMT of pancreatic cancer cells through activation of the ERK1/2 signaling pathway. In vivo experiments showed that knockdown of SEMA3C attenuates tumor growth, and overexpression of SEMA3C increases tumor size and weight [36]. In gliomas, SEMA3C expression is closely linked to their severity [18]. Hao et al. showed that combined inhibition of Wnt and SEMA3C pathways improves survival in a mouse model of glioblastoma [37]. Consistent with our results, it was shown that SEMA3C was significantly increased in TSCC, and its high expression was positively correlated with a poor prognosis. We also conducted a GO analysis of SEMA3C and found that SEMA3C was significantly enriched in BP regulation of cell proliferation (GO:0042127) and cell adhesion (GO:0007155). In our vitro experiments, we showed that deletion of SEMA3C in TSCC cells inhibited cell growth, migration, and invasion, consistent with the results of the previous study, indicating the feasibility of targeting SEMA3C therapy.

Our single-cell sequencing results showed that SEMA3C was mainly expressed in tumor cells. Greater than 90% of cells with SEMA3C expression in S1, S3, and S5 samples were tumor cells, while greater than 80% in S2, S4 samples, and 67.15% in S6 samples (due to the small number of tumor cells in the results). The above results suggest that targeting SEMA3C treatment has little side effect on other cells.

There were several notable shortcomings in our research. Firstly, although the research results were validated through in vitro experiments, in vivo confirmation was lacking, and further verification of these findings is needed in vivo studies. Secondly, the pathological mechanism of SEMA3C in the pathogenesis of TSCC is not yet clear, and further research is needed in the future.

## Conclusion

In this study, we preliminarily screened the key marker gene of TSCC and revealed for the first time that SEMA3C is involved in the development of TSCC. We demonstrated that SEMA3C deletion inhibits TSCC cells growth, migration, and invasion in vitro. Thus, SEMA3C might serve as an early clinical marker for TSCC.

### Supplementary Information

**Additional file 1: Table S1.** Tongue carcinoma-related dataset from the GEO database. **Table S2.** Differentially expressed genes between tongue carcinoma and control. **Table S3.** Genes identified by multiple machine-learning Strategies RF, LASSO, and WGCNA. **Table S4.** SEMA3C-related genes from the GSE31056 and GSE34105.

## Data Availability

The datasets used and/or analysed during the current study are available from the corresponding author on reasonable request.

## Abbreviations

TSCC: Tongue squamous cell carcinoma
GEO: Gene Expression Omnibus
ROC: Receiver operating characteristic
SEMA3C: Semaphorin3C
GO: Gene Ontology
ScRNA-seq: Single-cell RNA sequencing
ML: Machine learning
WGCNA: Weighted gene co-expression network analysis
LASSO: Least absolute shrinkage and selection operator
RF: Random forests
DEGs: Differentially expressed genes
KEGG: Kyoto Encyclopedia of Genes and Genomes
BP: Biological process
CC: Cellular component
MF: Molecular function
AUC: Area under the curve
PCA: Principal components analysis
UMAP: Uniform Manifold Approximation and Projection
qRT-PCR: Quantitative real-time polymerase chain reaction
DAB: 3,3′-Diaminobenzidine
IHC: Immunohistochemistry
CRPC: Castration‐resistant prostate cancer
HNSCC: Head and neck squamous cell carcinoma
OS: Overall survival
