# Supplementary material for: Integrated bioinformatics analysis of SEMA3C in tongue squamous cell carcinoma using machine-learning strategies
Source: Cancer Cell Int. 2024 Feb 6;24:58. doi: 10.1186/s12935-024-03247-y (PMC10845809; doi:10.1186/s12935-024-03247-y)
Supplement: Supplementary file 1 — Additional file 1: Table S1. Tongue carcinoma-related dataset from the GEO database. Table S2. Differentially expressed genes between tongue carcinoma and control. Table S3. Genes identified by multiple machine-learning Strategies RF, LASSO, and WGCNA. Table S4. SEMA3C-related genes from the GSE31056 and GSE34105. [file 12935_2024_3247_MOESM1_ESM.docx]

# Integrated Bioinformatics Analysis of SEMA3C in Tongue Squamous Cell Carcinoma Using Machine-learning Strategies

# Huixin Dou^1^, Can Song^2^, Xiaoyan Wang^1,3,4^, Zhien Feng^5^, Yingying Su^1*^, Hao Wang^1*^

^1^Department of Stomatology, Beijing Tiantan Hospital, Capital Medical University, Beijing, China

^2^Research and Development Department, Allife Medicine Inc., Beijing, China.

^3^Beijing Laboratory of Oral Health, Capital Medical University, Beijing, China

^4^Department of Biochemistry and Molecular Biology, School of Basic Medicine, Capital Medical University, Beijing, China

^5^ Department of Oral and Maxillofacial & Head and Neck Oncology, Beijing Stomatological Hospital, Capital Medical University, Beijing, China

*Corresponding Author: Hao Wang: Department of Stomatology, Beijing Tiantan Hospital, Capital Medical University, Beijing 100070, China. E-mail: [Hao_Wang2022@outlook.com](mailto:Hao_Wang2022@outlook.com)

Yingying Su: Department of Stomatology, Beijing Tiantan Hospital, Capital Medical University, Beijing 100070, China. E-mail: reliayysu@163.com

Table S1:

Tongue carcinoma-related dataset from the GEO database

| Dataset | Platform | Sample number | Tumor | Control | LN/ Margin |
| --- | --- | --- | --- | --- | --- |
| GSE31056 | GPL10526 | 96 | 23 | 24 | 49 |
| GSE34105 | GPL14951 | 78 | 62 | 16 | 0 |
| GSE160042 | GPL18180 | 20 | 10 | 10 | 0 |
| GSE13601 | GPL8300 | 58 | 31 | 26 | 1 |
| GSE138206 | GPL570 | 18 | 6 | 12 | 0 |
| GSE41613 | GPL570 | 97 | 97 | 0 | 0 |
| GSE172577 | GPL24676 | 6 | 6 | 0 | 0 |

Table S2:

Differentially expressed genes between tongue carcinoma and control

| DEGs | Gene symbol |
| --- | --- |
| Upregulated genes | ABL2, ADAM10, ADAM12, ADAM17, ADAMDEC1, AGRN, AIM2, ALDH1B1, ALPK2, ANGPT2, APCDD1L, APOC1, APOC2, APOL1, APP, ARL14, ARPC1B, ATP2C1, BIRC5, BMP1, BRMS1, BST2, C1QTNF6, CA9, CBS, CCL11, CCL5, CCNB1, CD300LF, CD38, CD86, CDC25C, CDCA3, CDKN2A, CLEC4A, CLEC7A, CLIC4, COL11A1, COL22A1, COL4A2, CSF2, CST1, CTHRC1, CXCL1, CXCL10, CXCL11, CXCL13, CXCL6, CXCL8, DCBLD1, DDX60, DDX60L, DIAPH3, DKK1, DLGAP5, DUSP10, EGFL6, ENPEP, EPSTI1, ESM1, EVA1A, F2RL2, FAM83A, FAT1, FBN2, FEZ1, FLNA, FN1, FOLR3, FST, GALNT10, GBP1, GBP5, GNLY, GREM1, GRP, HAS3, HERC5, HOXC4, HOXC6, HOXD10, IDO1, IFI27, IFI35, IFI44, IFI44L, IFI6, IFIT3, IL24, IL7R, INHBA, IRF1, IRF6, ISG15, ITGA3, ITGAX, ITGB4, ITGB6, KANK4, KIF23, KLHL5, KPNA2, LAMB3, LAMC2, LAMP3, LAPTM4B, LGALS3BP, LIMA1, LMNB1, LY6E, MAGED1, MARCKSL1, MDK, MFAP2, MICB, MISP, MMP1, MMP10, MMP11, MMP12, MMP13, MMP3, MMP7, MMP9, MND1, MSANTD3, MSLN, MUCL1, MYO1B, NAGS, NCAPD2, NEK2, NEK6, NFE2L3, NOX4, NRG1, NRIP3, NUP62, OAS1, OAS2, OAS3, OASL, ODC1, ODF2, OLR1, ORC6, PAEP, PDE7A, PDPN, PHLDB2, PI15, PKM, PLA2G7, PLAUR, PLEC, POPDC3, POSTN, PPP4R4, PSMB9, PSMC3IP, PTHLH, PTPDC1, PXDN, RAB23, RAB29, RAC2, RBM41, RBP1, RHPN2, RSAD2, SCG5, SDS, SERPINA1, **SEMA3C**, SHC1, SKAP2, SKIL, SLAMF8, SLC15A3, SLC16A10, SLC16A3, SLC30A1, SLC7A8, SLCO1B3, SLFN5, SMTN, SNAI2, SNX10, SOX11, SP110, SPATS2, SPECC1, SPOCD1, SPP1, SRGAP1, STAT1, STK17B, SULF2, TAP1, TINAGL1, TMEM44, TNFRSF12A, TNFSF10, TPM1, TREM1, TTPAL, TUBG1, TYMP, UBE2L6, WDR54, XAF1, XDH, XPR1, ZWINT |
| Downregulated genes | A2ML1, AADACL2, ABHD14B, ABHD5, ABI3BP, ABLIM1, ACAA2, ACKR1, ACP6, ACVR2A, ADGRG6, ADH7, AIF1L, ALDH3A2, ALOX12, ALS2CL, ANG, ANGPTL1, ANK2, ANKRD6, AOX1, APOD, ARHGAP32, ATOH8, ATP13A4, ATP6V0A4, ATP6V1C2, BAG1, BARX2, BBOX1, BEX2, BEX4, BICDL2, BNIPL, BOC, BSPRY, C10orf99, C11orf1, C12orf29, C15orf48, C1orf115, C1orf116, C3orf14, CAB39L, CAPNS2, CBR3, CCL14, CEACAM1, CEACAM7, CES2, CGNL1, CHCHD10, CHPT1, CIDEB, CILP, CIPC, CITED2, CLDN17, CLDN7, CLDN8, CLIC3, CLYBL, CNKSR3, COBL, COMP, COQ8A, COX7A1, COX7C, CRISP3, CRNN, CRTAC1, CSTB, CYB5A, CYBRD1, CYP2C18, CYP2E1, CYP2J2, CYP2R1, CYP3A5, CYP4F12, CYYR1, DAPL1, DEFB1, DEGS2, DLEU1, DNASE1L3, DPT, DUSP1, EBF1, ECHDC3, EFHD1, EIF4E3, ELF3, ELL3, EMP1, ENDOU, EPB41L4A-AS1, EPHB6, EPHX2, EPHX3, EPS8L1, ERBB3, ESPL1, ETNK2, EVA1C, EXPH5, FAM107A, FAM172A, FAM189A2, FAM3B, FAM3D, FGD5, FHL1, FLG, FMC1, FMO2, FMO3, FMO9P, FNDC4, FOXA1, FRAS1, FUT3, GBP6, GCNT3, GCOM1, GDF10, GDPD3, GGT6, GIPC2, GLTP, GPHN, GPRASP2, GREM2, GRHL3, GSN, GSTA4, GSTM4, GSTM5, HLF, HOPX, HPGD, ID2, ID4, IDNK, IL17D, IL1RN, IL36A, ISOC1, ITIH5, ITM2A, JAM2, KANK1, KAT2B, KIAA0232, KLF4, KLK12, KLK13, KRT13, KRT15, KRT4, KRT76, KRT80, KRTAP3-2, LDB2, LDOC1, LGI1, LPIN1, LRRN4CL, LY6G6C, LYPD3, MACC1, MACROD1, MAL, MAMDC2, MANSC1, MAOB, MAPK13, MCEE, ME3, MEIS1, MEIS2, MEOX2, METTL7A, MGLL, MGP, MGST2, MIPEP, MMRN1, MPP7, MPZL2, MSRA, MUC15, MXD1, MYH11, MYLIP, MYO5B, MYO5C, MYOC, MYRIP, N4BP2L1, NAP1L5, NCCRP1, NCOA1, NDNF, NEBL, NECTIN4, NFIA, NFIX, NMU, NUAK2, NUCB2, NYNRIN, PALMD, PARM1, PAX9, PCCA, PCOLCE2, PEG3, PGD, PGM5, PIGN, PIK3C2B, PIK3R1, PITX2, PLA2G4A, PLAGL1, PLBD1, PLEKHG6, PLLP, PLPP1, PLPP3, PLS1, PPARG, PPARGC1A, PPL, PPP1R3C, PRKX, PROS1, PRSS3, PTGR1, PTK6, RAB25, RALGPS1, RAVER2, RBP7, RDH12, REEP1, RERG, RGS5, RHCG, RNASE4, RNF175, RORA, RPS23, RSAD1, RSPO1, SAMD5, SAP18, SASH1, SCARA3, SCARA5, SCEL, SCIN, SCNN1B, SDCBP2, SERTAD4, SESN1, SETMAR, SFTA2, SGCG, SH3BGRL2, SH3GL3, SHE, SHMT1, SHROOM3, SIM2, SLC24A3, SLC25A25, SLC7A2, SLIT3, SLITRK5, SLURP1, SMAGP, SORBS2, SORT1, SPACA9, SPARCL1, SPINK5, SPINK7, SPNS2, SPRR2C, SRPX, STK39, SUSD4, SYBU, TACC1, TCP11L2, TF, TFPI, TGFBR3, TGM3, TGM5, TM7SF2, TMEFF2, TMEM178A, TMEM40, TMPRSS11B, TMPRSS11D, TMPRSS2, TP53INP2, TPRG1, TRIM29, TSPAN7, TSTD1, UBL3, UNC13B, VAV3, VIT, WASF3, XK, YOD1, ZBTB7C, ZFP3, ZNF185, ZNF273, ZNF395, ZNF415, ZNF750, ZSCAN16 |

DEGs, differentially expressed genes.

Table S3:

Genes identified by multiple machine-learning Strategies RF, LASSO, and WGCNA

| Strategy | Gene symbol |
| --- | --- |
| RF | SLC27A6, TNMD, EPB41L4A_AS1, FAM3B, PPARG, CAB39L, ID4, PTK7, EPHX2, FCER1A, RBP1, TCEAL2, TGFBI, INHBA, SUGCT, TROAP, VIT, TMPRSS11B, GTSE1, SAP18, TMEM184B, COL10A1, GPD1L, AURKA, GATM, CLU, ITM2A, PLAU, RBP7, **SEMA3C**, BMP1, LOC389328, DCBLD1, NMU, CYP4X1, TTYH3, ATP13A4, BIRC5, CCM2, CD302, CDK1, CEACAM1, CEP55, COL5A3, CYP4F12, CYSLTR1, DCLK1, DFNA5, DNMT3B, DTYMK, EN1, FAM221A, FNDC4, FSCN1, HJURP, HLF, HPGD, HSD17B6, IGSF10, ITGA3, KCNT2, KIF14, LRRC4C, LRRC8D, MAOB, MICAL2, MMP27, MYO1B, PITX2, PKMYT1, PLAC9, PLOD1, PMEPA1, PSMB2, SFTA2, SMDT1, SNAI2, SORBS1, SPP1, SSBP2, SUSD4, TCEA3, TF, TNC, TPBG, TRIP13, VAV3, WDR66, XPR1 |
| LASSO | SLC27A6, PTK7, OSR1, FAM3B, FAM107A, **SEMA3C**, GLDN, FCER1A |
| WGCNA | SPRR3, S100A9, CSTA, CSTB, ANXA1, B2M, RPS24, KRT4, RPL37A, HLA-A, SPRR1A, RPL23, TMSB10, NACAP1, MAL, LGALS3, NACA, HLA-B, ITM2B, GJB2, DBI, HOPX, CRNN, SPINK5, CNFN, HLA-C, EMP1, ATP5O, COL3A1, TUBA1B, CD9, BTF3, TGM3, PARK7, PSMA4, MT2A, SLPI, MYL12A, HLA-E, FAM162A, GBP1, SBSN, TMEM230, LUM, CXCL10, SOD1, SERPINB1, IVL, TMPRSS11B, NDUFB3, PSMB1, PRDX6, MAL2, HMGN3, COL6A3, DSG3, SAT1, LGALS1, SPINK7, TRMT112, TXNL1, CXCL9, COL1A2, RAB10, ALDH9A1, SLURP1, C10orf99, CAP1, PDLIM1, RHCG, DYNLT3, DEFB1, PPP1CC, S100A14, IFITM1, IGFBP7, AQP3, IFI16, PSMB4, SDR16C5, TGM1, PPL, NOP10, NCCRP1, CEACAM6, TM4SF1, NFE2L2, HLA-F, GSTO1, C15orf48, PRDX4, CHCHD2, LYPD3, CAPNS2, NMU, WFDC21P, PFN1, GSTA4, TNFSF10, COA3, MRPL36, THOC7, VPS26A, ADIRF, SUCLG1, RAB11A, FAM96A, HSBP1, ARF4, CAST, A2ML1, SNRPB, PSMB3, SRSF5, NUCB2, CMPK1, TSPYL1, ITGAV, ATP5G3, TGFBI, TUBB6, SDHD, TCEAL9, CEACAM5, TOB1, RAB25, CEBPB, UBE2L6, RPN2, SH3YL1, VDAC3, ZNF185, FNTA, MXRA5, SPARC, NPC2, GBP6, C9orf3, TMBIM6, SCEL, NFKBIZ, PSME1, ANXA5, ATP6AP2, GLTP, PSMD6, PTTG1IP, HEBP2, MSN, SERPINB2, CTSB, C14orf2, NT5C2, TRAM1, THBS2, TMED10, AIM1, EIF6, H3F3B, MICU2, OSTC, TAP1, EMC4, MALL, GOLPH3, KLF10, C5orf15, CLCA4, ECM1, HMGN4, RIN2, DDOST, PSMD8, DSC2, TMPRSS11D, SEC31A, DUSP6, BASP1, SELT, UBA3, MRPL14, PDIA6, HEXB, TNFAIP3, UBD, DSG1, CBR3, PSMA7, FTH1, VMP1, ARPC1B, RALGAPA1, FBXL3, VIMP, CDC123, CWC15, HSP90B1, IL1RN, PSMB6, IRF2BPL, MRPL27, FAM26F, VPS4B, SPRR2C, APEX1, TMED9, GPBP1, ZNF750, PTGR1, MTDH, SYPL1, MOB4, WARS, CIB1, CPNE3, BLNK, IFNGR1, RAB31, IGFL1, CXCL13, EMC7, SMAP1, UBE2I, PSMA5, LAPTM4B, PSMB9, HSD17B4, CNDP2, SS18L2, PALLD, PSMB8, MPZL2, PPP1CB, PHPT1, ARHGEF3, SUCLA2, SFT2D1, HLA-DQB2, PLBD1, SYF2, LYRM5, PELI1, GALNT1, MYCBP2, TXNDC12, MGST2, SACM1L, CRCT1, TOP2B, CTSC, SPG21, WSB2, EXOC1, NXN, SNAI2, SLC35F5, SUMO3, NELFE, MMP12, KLK11, TPD52L1, TRAPPC8, OBFC1, PSMD11, SERPINB13, LGALS3BP, LGALSL, LUC7L3, CAPG, SEC13, ACTN1, BST2, ALDH3A1, REXO2, METTL23, NUAK2, STXBP3, DNASE1L3, CNPY2, TSG101, KDELR2, PTBP1, ME1, FYTTD1, LASP1, PPP1R2, CTNNA1, TBCB, HIBCH, CCL5, SLK, SMIM10L1, AUP1, ADH7, EMP2, TMEM154, KLF4, TST, FAM49B, GBE1, MRFAP1L1, AMZ2, CLK1, COL1A1, DERL1, VCAN, LYPLAL1, INPP1, C17orf49, KLF5, ARL5A, ACKR3, PDCD6IP, LYRM1, DHRS1, EPS15, PPDPF, UBE2F, MXI1, FASTKD1, FRMD4B, IRF1, DHCR24, KLK12, SLTM, DERL2, ERO1A, KANK1, FHL2, PPP2R2A, TPRG1, LIMA1, RCBTB2, HK1, ATP5G1, SLC2A3P1, BIRC3, SHOC2, LAMB1, PSMB5, PNPLA8, ATP6V0B, ANKRD22, EIF2AK1, PLEKHA5, TNC, USP33, ACAA1, SLC9A3R1, SHISA5, ENY2, CDK4, FAM46B, ZHX1, POSTN, STAT1, GOLGA4, DSE, BTN3A2, UBE4A, RSRP1, PRPSAP1, CLPTM1L, PSMB10, FAM32A, DDIT4, PPP4R2, OXCT1, METAP1, EXOSC7, ACP5, GOLT1B, AIM2, FAM135A, MANF, INO80C, CXCL11, ARL1, MBOAT2, F2R, ODC1, KLK13, TM2D2, CTSA, SASH1, ALG2, CCDC12, HDGF, CRYZ, MYADM, RASGRP1, CES2, MATN2, PDCD4, ABLIM1, DUOX1, UROD, SMPDL3A, ID4, NIPSNAP3A, CPNE1, HDHD2, NONO, AATF, USP16, MRPS31, MORC3, C12orf29, METAP2, GLRX2, ARFGAP3, ADRB2, PRSS23, BBIP1, KRT78, CDK7, ACPP, RNF114, TMEM134, CLPX, ATXN10, MRPL17, ALDH3A2, PET100, LRRC59, ANKRD46, IL18, APOBEC3A, CCL4, COL5A2, UBE2G1, SUCO, ELF3, IL7R, ANKRD37, ECHDC2, RANBP9, PGM2, APP, SMAGP, RBMS1, APMAP, RAB5A, SNF8, YTHDF1, HMGB1, RAB34, MCEE, HINT2, FBXO3, ISOC1, ARL4C, CRELD2, AHSA1, VPS36, HCP5, EIF1AX, PNMA1, SON, NT5C3B, ELL2, STARD3NL, SULT2B1, KRCC1, NPEPPS, ITFG1, SDF4, DENND4C, SUMF1, COL4A1, ENDOU, RANBP6, IFI35, MMP9, KANSL1, IFRD1, KYAT3, SETD1B, CENPC, RNF111, TRAPPC3, MAGED1, MXD1, AFTPH, TRNP1, TBCC, TJP1, EIF2D, TMEM79, DRAP1, GALNT11, ZDHHC13, PFKP, LAMC1, TMEM203, CD1A, RNF135, WWP1, DMXL1, POLR2F, ADAMDEC1, AARS, TMEM251, CD207, LRRC1, LY6E, C21orf59, STK39, CRISP3, PTN, CLSTN1, BTN3A3, PRSS3, VKORC1, SEC23A, IDO1, UFL1, SLC35B1, FCGBP, GBP5, ADGRL2, TAB2, TMEM208, CLINT1, RSPRY1, ANKRA2, CALD1, PDCD7, ATP8B1, FN1, RALB, GON7, MGLL, TMEM263, GPX8, PEX11B, GRHL3, PSMC4, NANS, C9orf85, ENSA, MTCH2, HES1, UPP1, TMEM45B, DNAJB11, DNLZ, MAPK13, HEXA, PLA2G4A, KLHL5, UBR7, ATMIN, CYP2C18, APLP2, NARF, TUBG1, MIOS, TMEM138, SAE1, CFDP1, BOD1, PARP1, FAR1, SLC20A1, MRPS14, BAZ2B, GSTM3, CUL5, LRPAP1, TPST2, PITX1, LYSMD3, PCF11, CLIP4, CERS2, MEIS2, PEX3, SCNN1A, JOSD1, CCDC50, MAN2A1, XPA, CASP7, EIF5A, CCDC6, SNX10, NCK2, FSCN1, MYDGF, PPP1R18, LANCL1, CDS1, CRYBG3, AKTIP, PIM1, RTFDC1, RECQL, MVP, BFAR, AACS, MMD, PPID, ANKHD1, CNN2, ITPA, NLRC5, COX11, CROT, PTPN13, CXCL17, PNISR, PROCR, VSIG10L, ACOT9, SULF2, MNT, TNS3, MRPS16, ESRP2, NUAK1, CLDN4, ANKRD12, BEX2, ABCA1, ARV1, RUVBL1, P4HA2, PDLIM5, UBE3A, ZNFX1, LAGE3, USP4, GDI1, USP6NL, ABCD3, BCL2A1, NT5DC1, KCTD18, MYH9, MAOA, OSR2, TBC1D14, COL4A2, ZNF57, PLOD2, CMAS, C6orf203, ZNHIT6, POLR2C, SLU7, ZNF430, KIZ, CLOCK, N4BP2L2, PTK2, FBXO6, EXPH5, FBXO34, PHACTR2, ZDHHC3, ZNF721, NKTR, MTHFS, GHR, RPL31, MPZL1, RAB32, NRBF2, PITX2, ADO, TWSG1, LLPH, BBOX1, LCMT2, SLC3A2, POLR3K, COASY, ITGA5, PDE6D, POLE4, CTHRC1, SLC1A3, TLR3, EEF2K, MARVELD1, AHNAK, ZDHHC6, TRIM24, CHL1, GID8, WDYHV1, YAE1D1, SDF2, RAI14, ELOVL7, FAM83C, FBXO9, NKIRAS1, NCSTN, EIF2AK4, ASH1L, MRPL46, HSPA13, NUBP1, WDR26, UXS1, ASAP1, PM20D2, SNRPC, SLMAP, ARF3, YWHAH, STK17B, PMS1, PLSCR3, RIC8A, SEC61A1, GLB1, CLDN7, CD151, CAPN7, FUCA2, MFSD5, ZDHHC16, LONRF1, TGOLN2, MAN2B2, ABHD17C, GLA, SERPINB11, KLHL9, OXSR1, TMEM135, CHN1, PUS3, TXNL4A, MMP1, CBR4, CHST15, CHP1, PIK3CA, ATG101, SZRD1, MINA, SSR3, MCCC1, SREK1IP1, RAB40B, MFAP2, SPPL3, SOSTDC1, GLIPR1, CXCR2, NECAP2, DLAT, RGL2, CHMP1A, DNPEP, FADD, ALDH18A1, HACL1, SHPRH, HEXIM1, LYN, DRAM1, MTMR10, GCC2, GNA13, POLDIP2, ST14, ELOF1, ARL2BP, ST6GALNAC1, CDK2AP2, GMPR2, RAB11FIP1, LRBA, RSBN1L, CERS3, PLEKHM1, CYP2R1, G3BP2, SDCBP2, FAM13B, KIAA1468, TMEM218, MICB, DDRGK1, OGFRL1, RAB5C, ARGLU1, PRPS1, CRYZL1, SCAMP3, HEATR5B, WDFY1, COA5, CDKN2AIP, KATNBL1, TOR1A, CSNK1D, BMPR1A, WDR77, KIDINS220, CDH11, MINPP1, TRIM13, FAM101B, TMPRSS4, HDGFRP3, CLCN3, SMARCA5, FIG4, RNF38, USP7, COL16A1, EPC2, CHCHD4, FNDC3B, EVPL, SERPINH1, HSD17B8, TRIM33, BBX, BNIPL, PARP8, TMEM161B, AIP, VPS8, PRSS27, QRICH1, RFXANK, EPM2AIP1, RBM4, CWH43, CCM2, GRHL1, OXSM, NPLOC4, IREB2, LINC01420, APOC1, ZNF559, CLK4, FAM179B, CMIP, RBM6, BDKRB2, DMXL2, CAAP1, DLD, AGTPBP1, TRAPPC11, GTF2IRD1, NAXD, PDIA4, SMIM5, LHFPL2, CYB561A3, RWDD2B, TSPYL4, NSRP1, FAM172A, ALOX12, SNAP47, SLC11A2, AADAC, COL21A1, C1orf116, ATG4A, TM9SF1, RHOV, H2AFY2, TC2N, EPHA2, MPG, ADIPOR2, THY1, PELO, APPBP2, SECISBP2L, HLF, SNRNP35, PLOD3, GALNS, LPCAT1, NTMT1, SHC1, OSBPL2, NPAT, WBP4, YBEY, PDIK1L, PDXDC1, RCOR3, ZBTB5, SEPTIN9, EVA1C, SNX16, PART1, CALR, CRB3, FBXW7, RAPGEFL1, KCNK6, SKAP2, SMIM3, CUL3, RCBTB1, PLA2G12A, DLEU1, EFS, OVOL1, ALG3, GLTSCR1L, UNC13B, AKIP1, ACSS2, AUTS2, HPSE, XRN2, PPFIBP2, CDC27, ANPEP, LYRM7, RAB2B, CIPC, FAM126B, RNF169, PCOLCE2, SLC39A1, MGAT2, NMT2, CHST2, VLDLR, FAM84B, SPATA5L1, CEP76, ST5, CPPED1, PDGFC, FKBP15, CBFB, TFRC, PARD3, SEMA4B, SPATS2L, MFSD6, TMEM214, HIGD1A, TP53INP2, GCOM1, CD2BP2, CLIP1, ANKRD35, WDR7, ACOX1, CARD19, PRKCDBP, MAST4, EDEM2, NID2, DAP, USP47, ACP2, KDSR, TACC2, SLC25A12, RNF170, CPEB2, CPNE2, CCDC127, MOSPD1, PHF2, ZNF823, GPAA1, SLC35B2, FKBP1A, ZNF680, TRIOBP, CHAMP1, PCM1, MYO6, RAB24, DNAJC10, AAR2, RAB11FIP2, TXNRD1, SNRNP70, SLC1A5, ZFP62, TNIP2, ABHD5, IL15, NEDD4, PLOD1, GSE1, CALU, GPT2, STAU2, MT1F, TMEM168, DPY19L1, REV1, PDIA5, ENTPD3, MUS81, TADA1, BPTF, PUDP, MPV17, COPA, SLC48A1, LMO7, DIAPH1, ERMP1, EXTL2, EEF1AKMT1, MED10, FGFR3, B4GALT3, TRPS1, SGSM2, ARMCX5, FKBP14, ORMDL1, MRM3, MYO5C, EDNRA, AP1M1, NFYB, CIDEB, PHLDA1, DESI1, KIF9, RBP7, CNOT1, EHD3, MLEC, CDC42EP3, PRIMPOL, NEU1, TPM4, FAM98C, DFFA, SFTA2, TNFAIP6, FZD7, GDPD3, APOL6, CXCL1, ATP9A, TRIM47, LBH, TMEM40, HACE1, CDS2, PLAU, TM7SF2, CCDC25, TAF6, SCYL3, KIF1C, ELOVL4, TDRD3, HTRA2, ENDOD1, ABCA5, FAM63A, ZNF281, EML3, TRIM27, DIAPH2, TMOD3, B4GAT1, COL5A1, BAX, SLC38A6, ERBB3, GAREM1, EMC10, DPM2, TMEM41A, HOOK1, BTBD11, ELF4, USP53, SMARCA1, STRBP, GIGYF2, ANK3, TBC1D8, ASCC1, PAXIP1-AS1, EEFSEC, PNPLA4, ZNF212, TMED1, DHX16, ACTR3C, BID, TRIM35, SCFD2, SPON2, PLA2G7, MTFP1, DDX49, SIPA1L2, KPNA6, C4orf48, ZRANB1, ZNF43, CYP2J2, CNKSR3, TIAM1, DCAF10, CEACAM7, IRAK1, NOTCH2, DUSP10, RAB11FIP5, PRSS22, PYM1, CEP63, TMEM39A, COMMD5, WDR18, ARHGEF10L, SLC44A3, ZNF708, CLUH, USP46, HOOK2, DHX8, VAPA, GLMP, TCEB3, TLR2, TMEM246, PLCD1, ZNF518A, TMEM184B, ICK, USP25, SOCS6, TRIQK, IL4R, LRRC8C, VEGFC, RORA, GNA12, PRKX, SLFN5, KLF8, PGF, RPL23AP32, RHBDF2, RDH12, BSPRY, GMPPA, AXL, PEX12, EPS8L1, BRD4, TPPP, DDX19A, GTF3C5, CEACAM1, GRAMD1C, EEA1, HPCAL1, SART1, HLA-G, MTF1, PER2, SPNS1, EML4, RAET1E, C7orf50, ICOS, SCAPER, AGTRAP, RAB23, MTERF2, MEGF9, ANKRD6, CNPY3, SLC35C2, CTSZ, ALG9, SEPTIN11, DNAJC11, EVA1B, ACAD8, KRT80, SMAD7, ZNF395, PDIA3, PTPN1, GALNT2, HS1BP3, TMEM80, ARID4A, SPRYD4, TSPAN17, MAN1B1, GSTZ1, SOX4, TM9SF4, DOCK7, GOLM1, EPN2, HECTD4, MPP7, TGM5, RUFY3, YIF1B, P3H1, STAMBPL1, PYCR1, PPP2R5A, NID1, PSCA, LRRFIP2, HPS3, DUOXA1, SMYD3, BTN2A1, POF1B, FAM198B, ZNF654, SOAT1, TPST1, NELFA, PPOX, IFT22, YKT6, PIGN, ELK1, ATG13, ZNF529, NFKBIE, RHBDD2, CXorf38, SFT2D2, FAM160A2, RNF19B, EPHA1, MBOAT7, OSTM1, LARP6, FUK, B3GALT4, NCF2, VPS13D, UBFD1, SHKBP1, SEC24D, GLCE, TBC1D10B, RNPC3P1, PMEPA1, NPEPL1, CANT1, GNB4, MICAL2, ITPR2, PGAP3, ACOX3, FARP1, NCEH1, MYO5B, PTK6, RHOT2, TTC39A, SLC30A7, EPHX3, PHOSPHO2, CLASP2, TMCO3, LACTB, STX19, RNF40, MOV10, NLRX1, CTTNBP2, SGPP1, BCL2L13, MIR99AHG, DNASE2, PXDN, BCAT1, CARD10, NSMCE3, C8orf33, TFPT, SNX27, HERC1, LOC728061, GSDMD, MIPEP, CXCL8, VAC14, SC5D, PLPPR4, FLG, MEN1, ADPGK, FOXA1, LIN7B, MEIS1, FLRT2, KDELC1, UBAP2L, DVL3, SRPX2, JMJD7-PLA2G4B, NT5E, MLLT10, NTM, C11orf24, PAX9, CYB561D2, SPSB1, DEGS2, KDM2A, AMY2B, CACNA2D3, GLI3, RHBDD1, FADS1, HIRIP3, STOX2, MANBA, ATG7, SULF1, ALDH16A1, SLC9A1, EHD1, BIVM, GIPC2, PPP1R3D, RBKS, PANK1, EPN3, GBP4, MDK, ZC3H12A, ZDHHC21, PPP2R5D, TRAK1, UPK1A, SLC16A6, VPS13A, CHPF2, ARFRP1, PHYHD1, EYA2, ING3, PLS1, ZNF200, STX3, PRRC2A, SSSCA1, SLC16A3, LRP6, SLAIN2, NOTCH1, MMAA, SLC33A1, PSD3, SH3PXD2B, CLMP, ICAM1, KMT2C, PLAUR, ELL3, DHRS9, DBN1, H2AFX, PGM2L1, CSAD, TRAM2, ZADH2, TICAM2, CCDC146, ATP10B, SLC15A3, HYMAI, FLYWCH2, RPS6KA1, CYP4F12, HPS6, WDFY3, DGAT2, RMND5B, HSPA6, LAMA4, SPAG16, ADAM10, ZNF264, B3GALT6, FURIN, MRPL28, KRT23, BTN2A2, TCAIM, P2RY1, CNOT6L, SOX7, IKBIP, STIM1, EPHB6, B3GNT9, CHST11, FUT11, SLC7A1, NUP62, BRD8, TREM2, APOOL, TXNDC16, ERBB2, GPRASP1, STX2, PLEKHA7, CDK9, NEBL, CHSY3, ULK3, UBE2Q1, KLF13, BTN3A1, H2AFJ, EHF, PCBD2, SMPD2, SPACA9, MAGI3, COG7, SLC16A7, IDNK, USP54, DZIP3, MIIP, ADAT1, ALS2CL, ZFAND3, PPP1R13B, PDLIM4, TOX2, IGIP, MAP3K1, C12orf65, UGGT2, HMGXB3, DOHH, C2orf54, SPNS2, RBSN, SNX21, SERPINE1, SLC25A19, LINC01116, SH3D19, BCAR1, FAM219B, SH3GL3, IL17RD, FUT2, PATL1, TTC9, TNFRSF12A, ADA, ETV6, KIAA0232, SPATS2, FEZ1, GOSR2, ZNF552, SH2D4A, TSEN54, ATXN7, SMIM4, TMEM255A, INO80D, PRIMA1, P2RY2, TESK2, DDIT3, METTL21A, BATF2, SLC52A2, SOX2, ARHGAP27, ANGEL1, PADI1, ADGRG6, P4HA1, RAVER2, DSTYK, RCN3, SETDB2, COL6A1, ZNF514, C14orf28, THSD4, GPHN, ZNF350, SAP30L, ENGASE, AMN1, STARD3, THOP1, TMEM110, SYNGR1, NADK, LARP1, MUC15, NAT9, PARP16, WWTR1, RBM47, GPX7, HAUS7, HMGA1, NEDD1, BRWD1, NAA16, PRICKLE2, AS3MT, AHRR, TMC8, SNX8, HRASLS, SENP7, SLC25A22, FAM189A2, MAPKAPK2, RBM15B, TGFB1, ACP6, BMP7, TMEM2, MAU2, TRRAP, ZNF548, KRT31, TCF3, PCED1B, TATDN3, C15orf41, PCDH19, CYCS, MOB3A, TP53I13, CCDC88A, PLXNA2, SLC12A6, C16orf58, FZD2, ZNF502, P2RY6, KAZN, ANGPT1, SIAE, UBN2, NOXA1, AGAP1, LRWD1, MOB3C, ZNF555, PRODH, TMEM132C, GATSL3, ARHGAP32, ZC3H18, CEP126, TSHZ3, GALNT18, KIAA1211L, ESPL1, PTPN9, XK, SLC43A2, ZNF565, KMT2D, CNPY4, KCTD21, INO80B, FBXL16, DHX38, GGT6, NFE2L3, AIM1L, PDPN, LRG1, OPLAH, APH1B, MUL1, SPATC1L, AKAP9, TBL1X, TBRG4, **SEMA3C**, P3H4, CENPBD1, RGS14, C18orf25, LPAR5, CDIP1, ARSD, FNDC4, RHBDL2, DZIP1, LNX1, C15orf39, SEC22C, FAM83A, APBA3, ZNF204P, SLC39A13, C20orf27, SMU1, ZNF596, MID2, MFSD12, SLC39A7, PPFIA1, ABCG1, C15orf52, ZNF469, CXCL6, IL20RA, DAPK3, ZNF607, POLR3H, ANTXR1, SPATA2, NECTIN4, ZNF273, PTPRG, SMTN, ABCD1, SPHK1, CHST14, IL34, LOC441178, FAM207A, ZNF316, PRKAB2, TBC1D8B, GAB1, B4GALT7, LAMC3, KDELR3, LLGL2, ZNF253, ANO1, DDA1, AIF1L, PPP2CB, ZNF282, SHROOM3, GTDC1, L3MBTL3, NOC2L, MAB21L3, PELI2, CNTN3, MOGS, POLR3D, DOPEY2, ZNF879, LINC01588, ZNF248, DISP1, SUSD1, MTMR9LP, GUCY1B3, DTX2P1-UPK3BP1-PMS2P11, ARHGEF12, RNF121, FUT3, SALL2, RELB, FPGS, CCL20, MMP14, ZNF618, DLG3, ZNF317, EVI5, ATG4D, ENC1, PCDH17, YOD1, GATAD2B, RRN3P2, ZFP41, FAAH2, LOC653602, CDKN2D, MTSS1, GAS8, ZNF85, SGPP2, BICDL2, ZSCAN16, UBTD1, BCL9, ARHGEF17, SLC27A5, ZBTB7C, SCML2, AMT, MMP19, TUBBP5, GNLY, COL12A1, DAB2IP, SPOCK1, KLF11, TSPAN9, URB1-AS1, RMND5A, UST, PER3, RGAG4, NREP, PRSS3P2, TDO2, KANSL1L, SERAC1, DOCK9, FAXDC2, ZNF793, SIRPA, ECE1, ZNF485, CA13, SLCO4A1, LOC645513, ZNF439, FUT6, TRAM1L1, TEF, ZC3H6, SLITRK5, TDP1, SLC27A2, BCOR, CTLA4, LRRC20, CDK14, NEK6, PXN, KPNA5, CAPN15, SUCNR1, SNORA72, FZD1, FAAH, SAMD5, ARG1, SOX13, PHTF2, PLXNC1, EID2B, LOC390933, TAB3, SYTL4, CYP2E1, SPTSSB, REEP2, DMTN, RIPK4, ZNF681, GATA6, RELT, THRB, AKIRIN2, PLEKHG6, TMC6, ZNF789, SLC7A6, TRMT10A, ZNF792, SLC38A7, B3GNT8, TRIO, PGAP1, BMP1, UAP1L1, RPS6KA4, CHPF, ARFGAP1, NEIL1, ARHGAP23, ARHGEF9, PTRH1, C3orf58, ZBTB39, LRRC15, PI15, CRTAC1, TNFSF4, MEGF6, GLYR1, SCNN1B, SCRN3, UBE2O, PRR5L, GALNT10, LYRM9, ZHX3, CST4, SBSPON, PIK3CD, NTN1, TMEM259, TNFRSF10B, PRDM12, APBB2, LOC374443, TAPBP, CD38, WDR25, NPAS2, SORCS1, PAOX, HSF4, ZNF107, FUT4, POMT1, TRPC1, GXYLT1, AFAP1, APOBEC3F, CXCL3, NTRK2, CEP41, TOR2A, ITGB6, C1QTNF7, EMILIN1, ZNF554, PCDHB10, SLC35A2, ZDHHC4, ENPEP, FBLN7, ZNF493, ACTR3B, SPATA18, SMG5, TINCR, TAP2, MIER2, KMT2A, ETNK2, EPB41L4A-AS2, CAPN5, CD276, TSPAN2, HSD17B6, QPRT, FKBP7, FAP, TACSTD2, LOC647115, MAZ, TTYH3, ASPHD2, LSS, PITPNM1, HOXB2, RBM20, ARHGAP24, MBLAC2, LYNX1, SRGAP2, UBXN11, ARHGEF16, PTCHD3P1, F2RL2, FRAS1, LURAP1, APOM, ADAM8, RALGPS1, DACT1, CYP2W1, HEATR3, FSTL3, ZNF814, BCL2L11, NAT14, RAVER1, LEAP2, C21orf2, TFPI2, ADGRL1, CLHC1, ZNF566, TEX101, GLIS3, SIM2, SMOX, CSGALNACT2, NXPE3, EPB41L4A, ZNF677, CYP2C9, MMP7, ZNF135, LOC388780, FAM104B, TNFRSF9, SCLY, SCNN1G, ATP13A4, LEXM, GDF15, NUDT13, FGFBP2, DENND2C, ELFN1, SLC38A5, KIAA1644, SLC6A1, AGPAT4, ARID3A, ASB7, RGS4, REEP6, PCDH20, ROR2, HCG22, PADI3, TSPAN15, MACC1, LOC401052, SPP1, ZMAT1, NUDT16, ADORA1, DENND6B, NEGR1, LOC100129122, SEPTIN4, LOC644285, ALDH1B1, BEND6, ZBTB42, PHTF1, NPM2, ZNF711, ETV2, BRAT1, LEMD2, SLC13A4, FKBP10, C15orf59, PLEKHG2, LOC728613, TINAGL1, TREM1, CLEC11A, SGTA, KANK4, GDF11, MSC, RPL21P68, ADAMTS2, C3orf80, CPXM1, TMEFF1, MAPK8, COL10A1, ZNF662, ARMC9, CD70, NOX4, SEC14L2, SGIP1, APBA2, SHISA2, CLPB, MMP3, ACSM5, SNRPE, SEC14L1P1, LOXL2, TCP11L2, ALPK2, NOVA1, MBTPS2, DNAH5, SCG5, GPC6, PDCD1LG2, COL11A1, UNC93B1, PMEL, CCL11, THAP2, ZNF674-AS1, MYO3B, SLC41A2, SLC25A21, LOX, MATN3 |

RF, random forest; LASSO, Least absolute shrinkage and selection operator; WGCNA, Weighted gene co-expression network analysis;

Table S4:

SEMA3C-related genes from the GSE31056 and GSE34105

| Gene symbol |  |  |  |  |  |
| --- | --- | --- | --- | --- | --- |
| STT3A, RASD1, TNKS, MEST, ZNF438, SLC35B3, IGSF22, YTHDF3, CRYGC, HIPK1, RGMB, STX2, CLNK, RIPK1, CREB3L3, CCDC25, HMGB3, CDH4, DNA2, STX1A, LEP, H2AFV, KRT85, CDADC1, CNTD2, MEFV, CNKSR2, BANF2, DPEP3, ANO6, PCDHA9, MCM8, PNRC2, MNX1, PLK3, ANXA9, MED1, SLC6A15, VMO1, CCRL2, HRASLS2, CDC42EP2, CRYGN, SLC7A3, ZAR1, KLK6, CLUL1, SGCB, ZDHHC22, CIB3, TCFL5, PRR19, GCKR, ARC, PSD2, PIGK, ECHDC1, DKK3, HOXB4, KBTBD7, PLEKHA8, PLA2G12B, TRAIP, POU3F4, MCM3, FMO6P, PRAP1, BSND, MANBAL, MEX3A, TSSK1B, GPR26, AP4S1, GANAB, TIGD6, CPN2, ESRRB, MAP3K9, PRTN3, NR1I3, MVK, NOXO1, TMEM198, TEC, ZNF146, LMAN1L, OCIAD1, GJA9, OAZ3, DSN1, DHH, OR1D2, RAD1, GJC1, TNC, TINAG, GMPR2, COMMD6, STAT4, ONECUT3, COL8A2, EMX2OS, SIGLEC7, TBC1D5, NUDT17, NKAIN3, CA6, KLHL7, PRDM14, TRIM62, CEACAM4, SESN2, GP1BA, VSIG4, NEUROD6, ZBBX, COQ10B, SLC39A13, JMJD6, PSMA2, CARD16, DHX40, KDELR2, PRRT1, TRIM7, NFYB, SGPP2, AATK, PGLYRP3, DAP, MPST, PDGFC, CSHL1, LUC7L2, RNF139, C8G, NGDN, ISCA1, RLBP1, TREM1, FBXW5, YWHAZ, ARHGEF10L, FAM179B, NINJ1, SIRPD, HBEGF, HNF1A, PNPLA5, C15ORF62, DOLK, HMG20A, GRM4, SLC17A8, HYALP1, DHTKD1, CTRC, TRPM7, STAT3, AMIGO2, LRRCC1, MMP24, ZNF780B, SPEM1, GPR182, ZNF485, C21ORF62, CNTN6, CBX3, PGRMC1, PILRA, H1FOO, PHTF1, MTDH, ARHGEF2, NEK3, SERPINH1, COPS5, TMEM86A, DPF2, TOMM20, LENEP, SI, TAF1L, SMPDL3A, SCNN1G, KIF3B, KBTBD6, OCLM, DDI1, TAF1, ITIH2, OSBP2, GOPC, SEBOX, MYO1A, ZNF254, PCBD1, C11ORF57, TM4SF20, SAGE1, CFDP1, AMPD3, MTRF1, PRDM9, RBP2, GTF2I, SQRDL, SERPINB1, IDI1, MFSD6L, BIVM, ZDHHC14, BRMS1L, KBTBD2, ITPKA, UROC1, MYCBPAP, C1ORF35, TMEM53, CCL26, PAQR6, ZCCHC3, PTPDC1, LILRB5, MS4A7, CYP4A11, GSK3A, ACSS2, GZMB, TMEM19, CTRL, NDC80, AGK, TMEM86B, C11ORF16, BEND2, HAND2, C3ORF62, ZNF524, CAMK1, FDX1, PRTFDC1, KLHL17, CEACAM19, CCNE2, DCUN1D3, ARRDC4, CLSTN1, CENPJ, KRT25, CLDN14, VPS37D, FCER1A, TRH, TACR2, ADARB2, MPO, KRTAP9-3, CD248, KHDRBS1, C5ORF34, ARHGAP5, GLOD5, TMEM140, DEDD2, CARD14, CALCOCO2, ADAM10, OBFC1, HCRT, PCDH1, CASKIN1, TMEM147, PLAC8L1, MYBPC3, SUMF2, P2RX7, CASP4, WDR63, EPS8L3, STAT6, MATN3, C5ORF22, EPB41L3, HCST, KRT12, MN1, SERPINB8, METRNL, NEDD4, USH1C, NACC2, RNF20, ZNF507, TNFAIP2, RAB9A, GIGYF1, MLN, NBR2, ZNF362, CCDC60, ALAS1, ADAMTSL5, FLRT2, GNA12, AXL, FCER1G, SIGLEC5 | | | | | |
